# Spontaneous Decomposition of an Extraordinarily Twisted and *Trans*‐Bent Fully‐Phosphanyl‐Substituted Digermene to an Unusual Ge^I^ Cluster

**DOI:** 10.1002/anie.202208851

**Published:** 2022-08-25

**Authors:** Keith Izod, Mo Liu, Peter Evans, Corinne Wills, Casey M. Dixon, Paul G. Waddell, Michael R. Probert

**Affiliations:** ^1^ Main Group Chemistry Laboratories School of Chemistry Newcastle University Newcastle upon Tyne NE1 7RU UK; ^2^ School of Chemistry Newcastle University Newcastle upon Tyne NE1 7RU UK

**Keywords:** Cluster, Germanium, Multiple Bond, Phosphorus, Solid-State Structure

## Abstract

Ditetrelenes R_2_E=ER_2_ (E=Si, Ge, Sn, Pb) substituted by multiple N/P/O/S‐donor groups are extremely rare due to their propensity to disaggregate into their tetrylene monomers R_2_E. We report the synthesis of the first fully phosphanyl‐substituted digermene {(Mes)_2_P}_2_Ge=Ge{P(Mes)_2_}_2_ (**3**, Mes=2,4,6‐Me_3_C_6_H_2_), which adopts a highly unusual structure in the solid state, that is both strongly *trans*‐bent and highly twisted. Variable‐temperature ^31^P{^1^H} NMR spectroscopy suggests that **3** persists in solution, but is subject to a dynamic equilibrium between two conformations, which have different geometries about the Ge=Ge bond (twisted/non‐twisted) due to a difference in the nature of their π‐stacking interactions. Compound **3** undergoes unprecedented, spontaneous decomposition in solution to give a unique Ge^I^ cluster {(Mes)_2_P}_4_Ge_4_⋅5 CyMe (**7**).

## Graphical abstract

The first example of a fully phosphanyl‐substituted digermene, {(Mes)_2_P}_2_Ge=Ge{P(Mes)_2_}_2_ (**3**), exhibits a uniquely distorted Ge=Ge double bond. Unexpectedly, **3** is unstable in solution, decomposing to a novel Ge^I^ cluster (**7**).

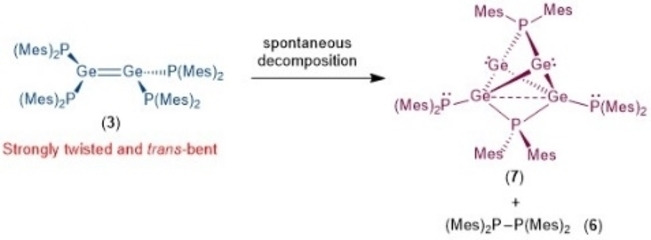

## Introduction

Compounds containing highly distorted (i.e. bent, twisted) element‐element double bonds continue to be of intense interest, both from a theoretical perspective and due to their unusual physical and chemical properties. For the C=C double bond in alkenes numerous strategies have been devised to induce these distortions.^[1]^ Frequently these involve the synthesis of sterically overcrowded alkenes, for which repulsion between the substituents at either end of the double bond disfavors planarity at the alkene carbon atoms and/or leads to a twist around the C=C bond. At the extreme twisting limit, where the two ends of the alkene are orthogonal, π‐overlap is minimal and diradicaloid character may be expected.^[2]^

**Figure 1:**
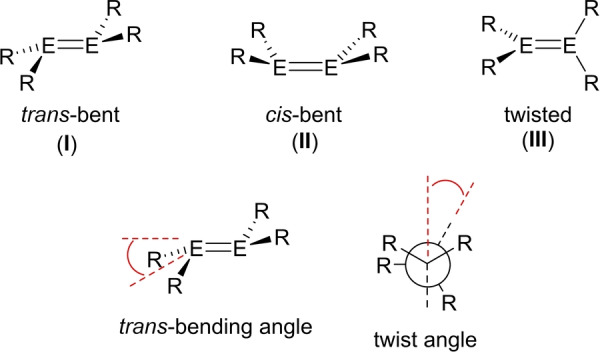
Typical conformations of ditetrelenes (E=Si, Ge, Sn, Pb) and definition of *trans*‐bending and twist angles in *trans*‐bent ditetrelenes.

For the heavier group 14 analogues of alkenes (ditetrelenes, R_2_E=ER_2_, E=Si, Ge, Sn, Pb), such distortions are the norm, rather than the exception and these compounds typically adopt a *trans*‐bent structure in the solid state (**I**, Figure 1).^[3]^ The non‐planar structures of these compounds may be rationalized on the basis of a second‐order Jahn–Teller effect involving mixing of the E=E π‐ and σ*‐orbitals in the *trans*‐bent geometry.[^3^, ^4^] This leads to stabilization of the HOMO, which may be described as a slipped π‐bond with significant non‐bonding electron density at the element centers.

While a *trans*‐bent geometry is commonly observed in ditetrelenes, compounds with alternative *cis*‐bent (**II**) and twisted (**III**) geometries have occasionally been isolated.[^3^, ^5^, ^6^, ^7a^] The adoption of these alternative geometries has been attributed to steric effects (i.e. repulsion between bulky substituents at the tetrel centers) and/or crystal packing forces. For ditetrelenes, strategies to induce a particular distortion are limited and there is little understanding of the factors which influence the prevalence of one type of distortion over another.

**Figure 2:**
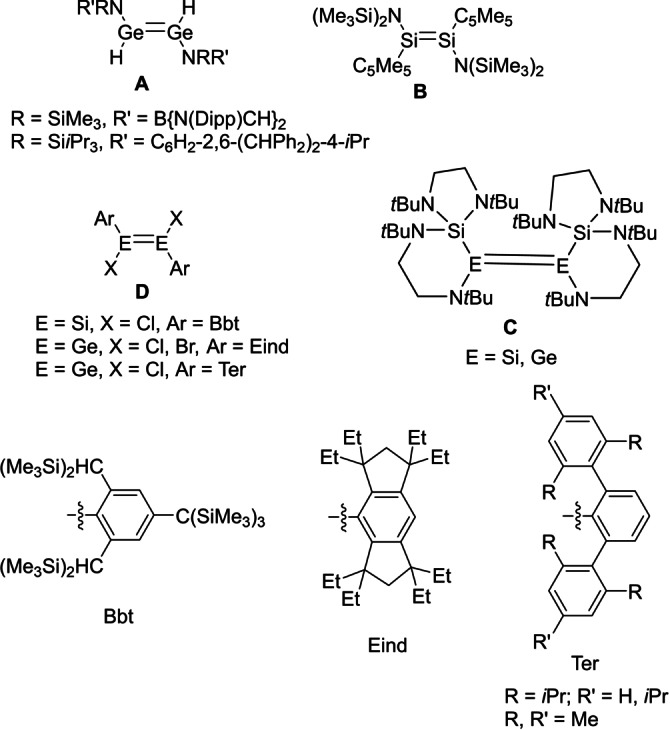
Selected examples of disilenes and digermenes bearing multiple heteroatom substituents (other than Si).

While the chemistry of ditetrelenes is well established, examples are largely limited to aryl‐ and/or silyl‐substituted species.^[3]^ Few ditetrelenes bearing heteroatoms other than silicon have been isolated, with such compounds usually dissociating to the corresponding tetrylenes R_2_E:. Indeed, until recently, only a handful of compounds containing two such substituents were known (Figure 2) and no compounds containing three or more heteroatom substituents had been isolated.[^7^, ^8^] In this regard, although in solution a dynamic equilibrium has been proposed between the diaminosilylene (*i*Pr_2_N)_2_Si: and its disilene dimer (*i*Pr_2_N)_2_Si=Si(N*i*Pr_2_)_2_ (**1**), based on variable‐temperature UV/Visible spectroscopy and DFT calculations,^[8]^ the proportion of **1** was found to be very small, even at low temperatures, and this compound has not been isolated in the solid state.

**Figure 3:**
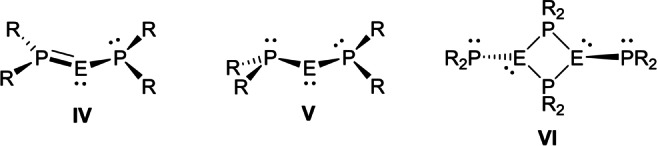
Structures of diphosphatetrylenes (R_2_P)_2_E.

The reluctance of O/N/S‐substituted tetrylenes R_2_E: to dimerize to the corresponding ditetrelenes R_2_E=ER_2_ may be attributed to the efficient stabilization of the former through O/N/S−E π‐interactions.^[9]^ For phosphanyl‐substituted tetrylenes, (R_2_P)_2_E:, P−E π‐interactions are observed only in a very small number of sterically hindered compounds (**IV**, Figure 3),^[10]^ due to the significant barrier to planarization at phosphorus, which typically favors compounds that are free from such π‐interactions (**V**).^[11]^ With less hindered ligands, phosphanyl‐bridged dimers (R_2_P)E(μ‐PR_2_)_2_E(PR_2_) (**VI**) are isolated.^[12]^

In spite of the foregoing, we have recently shown that a careful choice of substituent at phosphorus enables the isolation of a fully‐phosphanyl‐substituted disilene {(Mes)_2_P}_2_Si=Si{P(Mes)_2_}_2_ (**2**),^[13]^ the first example of a fully heteroatom‐substituted ditetrelene in which the heteroatoms possess a lone pair (Mes=2,4,6‐Me_3_C_6_H_2_). Unfortunately, **2** proved insoluble, even in strong donor solvents such as THF, preventing a detailed study of its reactivity.

In an effort to expand this series of compounds and to elucidate their reactivities, we have begun to explore the synthesis of related phosphanyl‐substituted ditetrelenes. Herein we report the isolation of the germanium analogue of **2**, which exhibits a highly unusual solid state structure, and show that this compound undergoes unprecedented, spontaneous decomposition to a unique Ge^I^ cluster.

## Results and Discussion

**Scheme 1:**
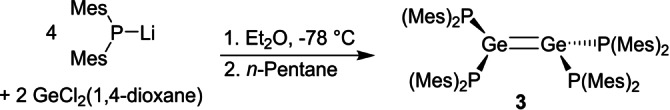
Synthesis of **3**.

The reaction between GeCl_2_(1,4‐dioxane) and two equivalents of [(Mes)_2_P]Li in cold diethyl ether gave a deep blue solution. Removal of the solvent, while maintaining the temperature below −40 °C, gave a dark blue, sticky solid, which was extracted into cold *n*‐pentane and crystallized as dark blue blocks of the fully phosphanyl‐substituted digermene {(Mes)_2_P}_2_Ge=Ge{P(Mes)_2_}_2_ (**3**) (Scheme 1). In contrast to the insoluble disilene congener **2**, digermene **3** has good solubility, even in hydrocarbon solvents such as *n*‐pentane and toluene. However, dissolution of **3** in these solvents results in gradual decomposition at room temperature (see below).

**Figure 4:**
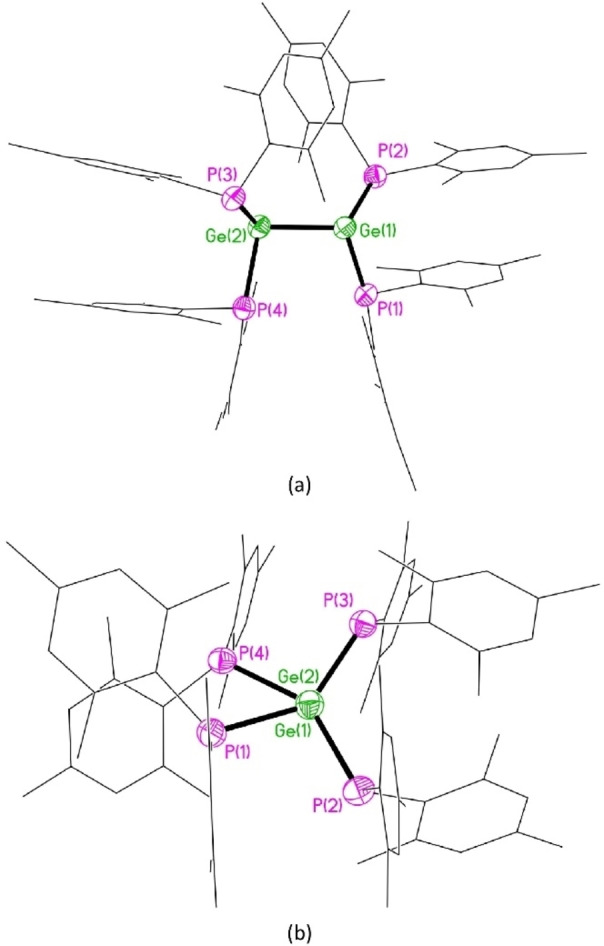
Molecular structure of one of the two independent molecules of **3** viewed a) perpendicular to, and b) along the Ge−Ge axis; H atoms and solvent of crystallization omitted for clarity. Selected bond lengths [Å] and angles [°] [values for the second independent molecule in the asymmetric unit in square brackets]: Ge(1)‐Ge(2) 2.4476(9) [Ge(3)‐Ge(4) 2.4701(9)], Ge(1)‐P(1) 2.3318(16) [Ge(3)‐P(5) 2.3371(18)], Ge(1)‐P(2) 2.3176(16) [Ge(3)‐P(6) 2.3540(17)], Ge(2)‐P(3) 2.3483(15) [Ge(4)‐P(7) 2.3619(18)], Ge(2)‐P(4) 2.3753(16) [Ge(4)‐P(8) 2.3428(15)], P(1)‐Ge(1)‐P(2) 104.54(5) [P(5)‐Ge(3)‐P(6) 98.35(6)], P(3)‐Ge(2)‐P(4) 95.58(5) [P(7)‐Ge(4)‐P(8) 96.78(4)].

**Figure 5:**
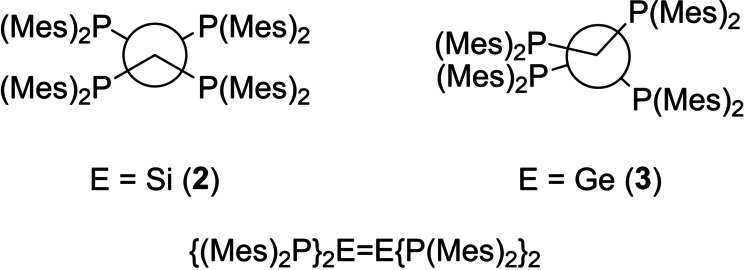
Newman projections illustrating the different conformations of the E_2_P_4_ cores of **2** and **3**.

X‐ray crystallography reveals that **3** has a strikingly different structure in the solid state compared to that of **2** (Figures 4 and 5).^[14]^ While both compounds are substantially *trans*‐bent, **3** exhibits significantly greater deviation from planarity at the tetrel center (*trans*‐bending angles 65.9 and 71.2° [62.9 and 74.6°] for each end of the molecule in **3**, versus 40.6° in **2** [values in square brackets refer to the second independent molecule in the asymmetric unit]). The *trans*‐bending angles in **3** represent the largest observed in any crystallographically characterized digermene; for comparison, the largest previously reported *trans*‐bending angles of 41.3 and 42.3° were observed in the unusual digermene (*Z*)‐**C_Ge_**.^[7a]^

**Figure 6:**
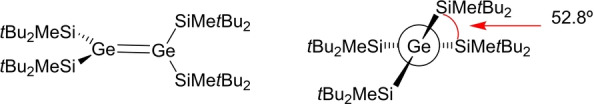
The twisted, but non‐*trans*‐bent digermene **4** viewed perpendicular to (left) and along (right) the Ge−Ge direction.

In addition, whereas **2** possesses an inversion center midway along the Si−Si vector, **3** is highly twisted (twist angle 44.85° [41.50°]). These twist angles are second only in magnitude to that of the blue digermene (*t*Bu_2_MeSi)_2_Ge=Ge(SiMe*t*Bu_2_)_2_ (**4**, Figure 6), which has a twist angle of 52.8°, but which possesses planar Ge centers and so is not *trans*‐bent.[^6f^, ^15^] The combination of a large *trans*‐bending angle and a significant twist angle leads to a configuration in which two of the phosphorus atoms in **3** approach an eclipsed conformation (P(1)‐Ge(1)‐Ge(2)‐P(4) dihedral angle 41.81(5)° [P(6)‐Ge(3)‐Ge(4)‐P(7) 37.10(6)°]). This places two of the phosphorus centers in close proximity (P(1)⋅⋅⋅P(4) 3.7712(19) Å [P(6)⋅⋅⋅P(7) 3.566(2) Å]); the shorter of these P⋅⋅⋅P distances lies within the sum of the van der Waal's radii for two P atoms (3.6 Å). Remarkably, the P_2_Ge=GeP_2_ core of the solid state structure of **3** bears a close resemblance to the N_2_Si=SiN_2_ core of the calculated structure of the tetraaminodisilene **1**, which is predicted to possess a twisted, *trans*‐bent geometry, with a *trans*‐bending angle of 42.6°, and a twist angle of 55°.^[8b]^

Twisting in ditetrelenes is usually associated with sterically bulky substituents. However, in the present case the non‐twisted disilene **2** and the twisted digermene **3** possess identical substituents and so should possess similar steric properties. Indeed, the slightly greater covalent radius of Ge^II^ (1.20(4) Å) compared to Si^II^ (1.11(2) Å)^[16]^ should lead to longer Ge−Ge and Ge−P distances and so might be expected to *decrease* steric compression about the tetrel centers in **3** compared to **2**. Thus, an alternative explanation is required for the different structures of **2** and **3**.

**Figure 7:**
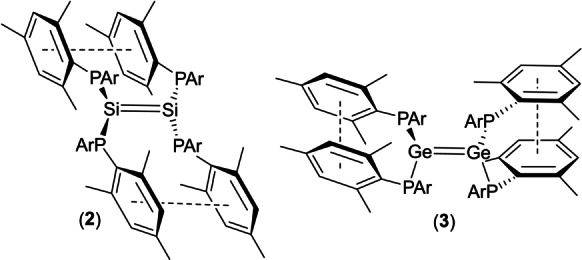
Illustration of the different π‐stacking modes in **2** and **3** (Ar=Mes).

To this end, inspection of the solid‐state structures of these two compounds reveals a difference in π‐stacking mode of the aromatic rings. In the non‐twisted disilene **2**, the mesityl rings are positioned such that there are π‐stacking interactions between rings which span the Si=Si bond (Figure 7). In contrast, in the twisted digermene **3** these interactions are replaced by π‐stacking interactions between mesityl rings associated with the same germanium center. Thus, in **2** the π‐stacking interactions act to lock the disilene into a non‐twisted geometry, while in **3** the π‐stacking interactions leave the molecule free to rotate about the putative Ge=Ge bond.

The Ge−Ge distance in **3** (2.4476(9) [2.4702(8) Å]) is at the longer end of the range of reported Ge=Ge distances,^[3]^ consistent with a weaker bond due to poor Ge=Ge π‐overlap in such a highly twisted digermene. However, similar Ge−Ge distances have been observed in non‐twisted digermenes which contain two substituents bearing a lone pair of electrons. For example, the Ge−Ge distances in the *trans*‐bent, but non‐twisted compounds (*E*)‐Ter(Cl)Ge=Ge(Cl)Ter and (*E*)‐Bbt(Br)Ge=Ge(Br)Bbt are 2.443(2) and 2.5087(7) Å, respectively [Ter=2,6‐(2,4,6‐Me_3_C_6_H_2_)C_6_H_3_; Bbt=2,6‐{(Me_3_Si)_2_CH}_2_‐4‐{(Me_3_Si)_3_C}C_6_H_2_];[^7e^, ^7f^] the Ge−Ge distance in the diaminodigermene (*Z*)‐**C_Ge_** is 2.460(1) Å.^[7a]^ In fact, the Ge−Ge distance in **3** falls within the range of typical Ge−Ge single bond lengths; for example, the Ge−Ge distance in (*p*‐MeC_6_H_4_)_3_Ge‐GeMe_3_ is 2.4292(7) Å,^[17]^ while the Ge−Ge distances in (Ph_3_Ge)_4_Ge range from 2.4842(6) to 2.5136(6) Å.^[18]^ The Ge−P distances span the range 2.3176(16)–2.3619(18) Å, which is typical for Ge^II^‐P single bonds.^[11a]^

**Figure 8:**
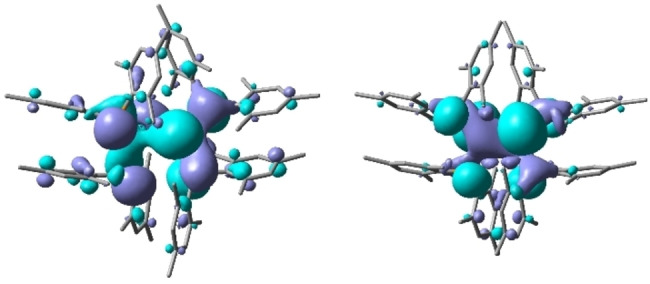
HOMO (left) and LUMO (right) of **3**.

DFT calculations on **3**, at the B97D/6‐311G(2d,p) level of theory, yield a geometry which closely resembles the solid‐state structure. In particular, the severe *trans*‐bending and twist angles and the π‐stacking interactions observed in the solid state are well reproduced in the calculated structure, although the Ge−Ge distance is overestimated by approximately 0.09 Å (see the Supporting Information). Natural Bond Orbital (NBO) analysis indicates limited multiple bond character in the Ge−Ge bond [Wiberg bond index (WBI) 1.011], most likely due to the substantial twist angle, which prevents the correct alignment of the germanium orbitals for efficient π‐overlap. In line with this, the HOMO of **3** is not a straightforward Ge−Ge π‐type orbital, but has a significant component of both Ge and P lone pair character (Figure 8).

The solid‐state CP‐MAS ^31^P{^1^H} NMR spectrum of **3** exhibits poorly resolved multiplets at 21.6 and −28.2 ppm, consistent with the solid‐state structure; these chemical shifts are substantially different from those exhibited by **2** in the solid state (−55.9 and −77.9 ppm), in accord with the significantly different structures of these two compounds.

**Figure 9:**
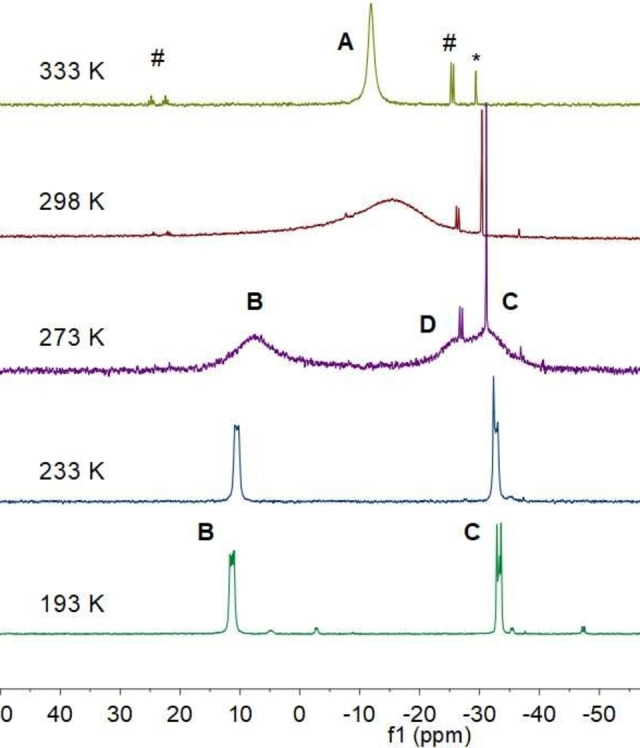
Variable‐temperature ^31^P{^1^H} NMR spectra of **3** in *d*_8_‐toluene [*(Mes)_2_P−P(Mes)_2_ (**6**), # signals due to decomposition product **7** (see below)].

In contrast to the solid‐state spectrum, the room temperature, solution state ^31^P{^1^H} NMR spectrum of **3** in *d*_8_‐toluene consists of a single, very broad singlet centered at −16 ppm (FWHM approx. 3000 Hz; A, Figure 9), indicating equivalence of the phosphorus centers on the NMR time scale at this temperature. As the temperature is reduced, this peak broadens and decoalesces, until, at 273 K, the spectrum consists of a pair of approximately equal intensity, broad singlets at 8 and −31 ppm (B and C, respectively), along with a third, broad singlet at approximately ‐27 ppm (D). As the temperature is reduced further, peak D decreases in intensity and peaks B and C sharpen, until, at 193 K, the spectrum consists of a pair of multiplets at 11.4 and −32.4 ppm, corresponding to an AA′BB′ spin system, consistent with effective local *C*_2_ symmetry of the Ge_2_P_4_ core at this temperature.^[19]^ A ^31^P{^1^H}‐EXSY spectrum of **3** at 263 K indicates exchange among all three peaks (B, C, and D) at this temperature.

**Scheme 2:**
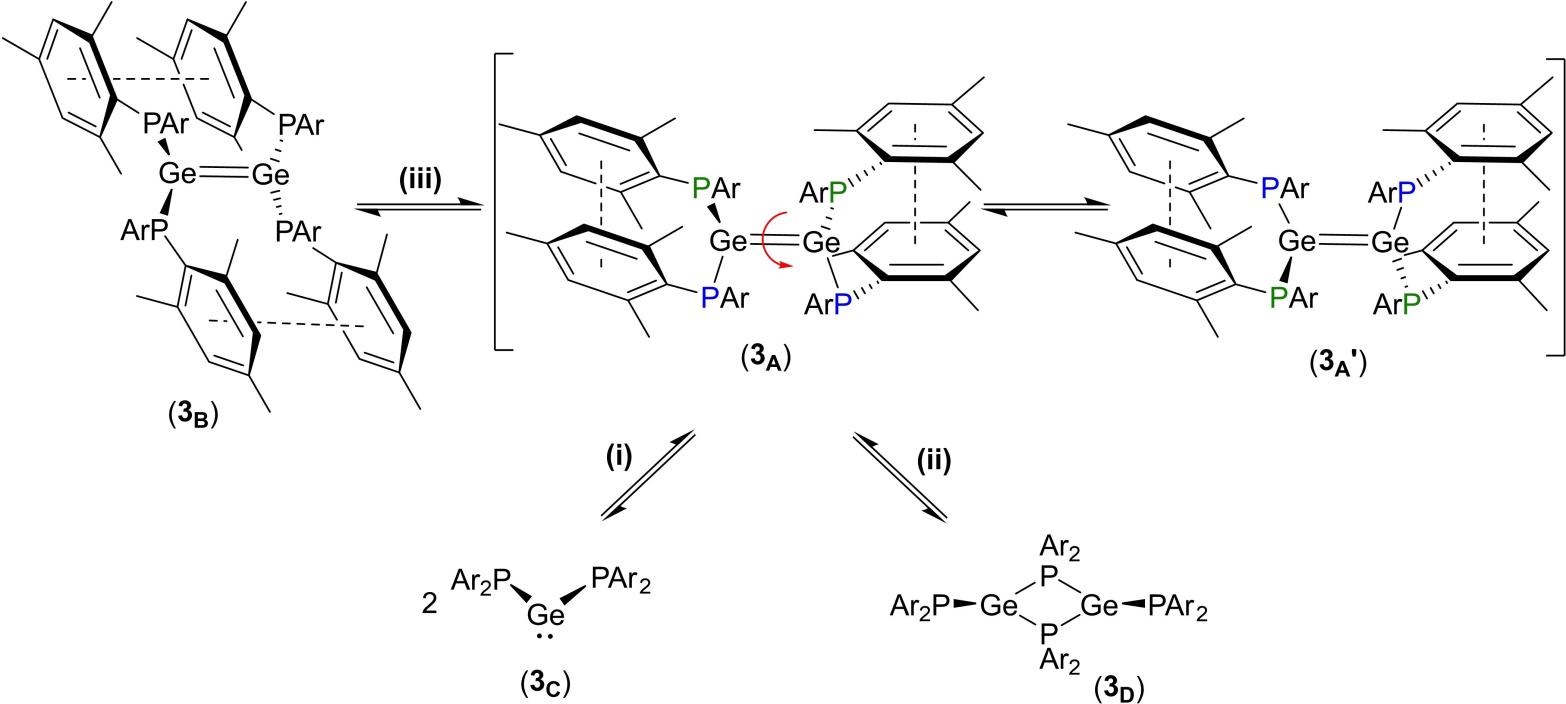
Possible dynamic equilibria for **3** in solution (Ar=Mes).

The low‐temperature ^31^P{^1^H} NMR spectrum of **3** is consistent with both the highly twisted, *trans*‐bent solid‐state structure of this compound and its solid‐state ^31^P{^1^H} NMR spectrum, while coalescence of the peaks at room temperature is consistent with rapid rotation about the Ge=Ge bond, which would lead to equivalence of the P environments at high temperatures. However, the observation of a third peak (D) at 273 K indicates that a second dynamic process must be in operation. This process may be (i) an equilibrium between the twisted digermene **3_A_** and two equivalents of germylene {(Mes)_2_P}_2_Ge (**3_C_**, Scheme 2), (ii) an equilibrium between **3_A_** and a phosphanide‐bridged dimer (**3_D_**), or (iii) an equilibrium between the twisted (**3_A_**) and non‐twisted (**3_B_**) forms of **3** (i.e., exchange between the two π‐stacking modes).

For process (i) DFT calculations indicate that the free energy of dissociation of **3_A_** into its constituent germylene fragments **3_C_** is +106.7 kJ mol^−1^, suggesting that the dynamic equilibrium observed for **3** does not involve this process. In support of this, we note that, while dissociation of digermenes into their germylene constituents has been observed previously for both silyl‐ and aryl‐substituted digermenes, the twisted (but not *trans*‐bent) digermene **4** remains intact in solution at room temperature, according to variable‐temperature UV/Visible spectroscopic experiments.^[6f]^

Digermenes and germylenes typically afford different products on reaction with unsaturated compounds and silanes, and so such reactions should enable us to confirm whether the germylene **3_C_** is present in solution. However, addition of any of 2,3‐dimethyl‐1,3‐butadiene, 1,2‐bis(trimethylsilyl)ethyne or Et_3_SiH to a solution of **3** merely led to rapid decomposition, with the diphosphane (Mes)_2_P−P(Mes)_2_ as the only identifiable phosphorus‐containing product (see below for further discussion of this decomposition). Attempts to estimate the hydrodynamic radii of the species present in solution at 273 K by ^31^P DOSY NMR spectroscopy were frustrated by the short relaxation times of the nuclei, which make them inappropriate to study by this method.

For process (ii), isomer **3_D_** is calculated to lie 61.6 kJ mol^−1^ higher in free energy than the twisted digermene ground state **3_A_**. Although the phosphanide‐bridged dimeric form **3_D_** would be expected to exhibit two sets of triplets in its ^31^P{^1^H} NMR spectrum, rapid exchange between the bridging and terminal phosphanide ligands at 273 K may lead to a time‐averaged signal, consistent with the observed signal D. However, it has been shown that the phosphanide‐bridged dimers (*i*Pr_2_P)Ge(μ‐*i*Pr_2_P)_2_Ge(P*i*Pr_2_)^[12d]^ and (PhRP)Ge(μ‐PPhR)_2_Ge(PPhR) (R=(Me_3_Si)_2_CH),^[12e]^ the only two such species to have been crystallographically characterized, do not undergo exchange of the bridging and terminal phosphanide ligands at room temperature.

Finally, for process (iii), DFT calculations reveal that the twisted form **3_A_** is 44.6 kJ mol^−1^ lower in free energy than the corresponding non‐twisted form **3_B_**. Given the foregoing, while we cannot, with the available data, completely rule out process (ii), we tentatively ascribe the observed process to a dynamic equilibrium between the dimers **3_A_** and **3_B_** (i.e. process (iii)), which operates in tandem with rotation about the Ge−Ge bond in form **3_A_**.

Prior to this study, the twisted conformation of a small number of ditetrelenes had been confirmed by X‐ray crystallography in the solid state, while the retention of this conformation for **4** in solution had been inferred from variable‐temperature UV/Visible spectroscopic measurements.^[6f]^ In the present case, the observation of an AA′BB′ spin system for **3**, and the correspondence between the solid‐state and low temperature solution ^31^P{^1^H} NMR spectra of this compound, strongly suggest that its twisted conformation is retained in solution at low temperature, providing NMR spectroscopic evidence for the twisting of a ditetrelene for the first time.

Although digermene **3** is stable for several weeks at room temperature in the solid state, storage of a toluene solution at room temperature resulted in a color change from deep blue to dark red over a period of several days, suggesting complete decomposition over this period. This apparent decomposition is accelerated in donor solvents: storage of a solution of **3** in diethyl ether in the absence of light led to complete loss of its blue color after 2 days, whereas this process was complete within hours in THF; this color change was also accelerated by exposure to daylight.

**Figure 10:**
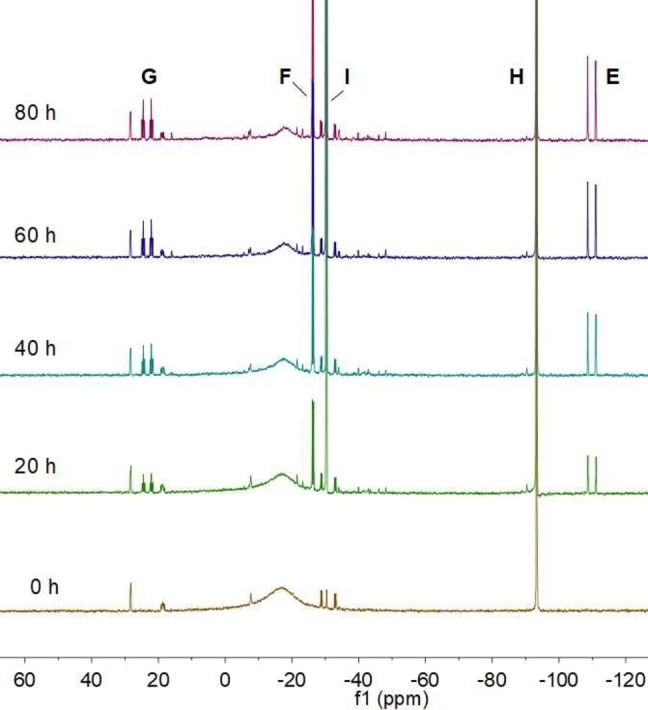
^31^P{^1^H} NMR spectra of a solution of **3** in toluene recorded at 20 h intervals.

The decomposition of **3** may conveniently be monitored by ^31^P{^1^H} NMR spectroscopy (Figure 10). This revealed that the broad singlet at −16 ppm due to **3** is gradually replaced by a pair of doublets at −110.9 (E, *J*_PP_=485.9 Hz) and −26.3 ppm (F, *J*_PP_=79.0 Hz) and a doublet of triplets at 23.2 ppm (G, *J*_PP_=485.9 and 79.0 Hz) in a 1 : 2 : 1 ratio, corresponding to an AM_2_X spin system. In addition, singlets at −94.5 (H) and −30.8 ppm (I), due to (Mes)_2_PH (**5**) and (Mes)_2_P−P(Mes)_2_ (**6**), respectively, are apparent, the latter peak also gradually increasing in intensity with time. Further monitoring of the solution revealed that peaks E, F, and G reach a maximum and then steadily decrease in intensity, eventually leaving only peaks H and I due to **5** and **6**. It therefore appears that the species giving rise to peaks E, F, and G is a short‐lived intermediate on the decomposition pathway of **3**.

The identity of this red intermediate was finally revealed by X‐ray crystallography. Deep red single crystals of the Ge^I^ cluster {(Mes)_2_P}_4_Ge_4_⋅5 CyMe (**7**) were obtained by recrystallization of the intermediate species from methylcyclohexane. Unfortunately, it was not possible to isolate a bulk sample of **7** in high purity due to its continuing decomposition, which generates **6** as a side‐product, and to the similar solubilities of **6** and **7**. Attempts to separate **6** and **7** by column chromatography or size‐exclusion chromatography under anaerobic conditions resulted only in rapid decomposition of **7** to **5** and **6**.

**Figure 11:**
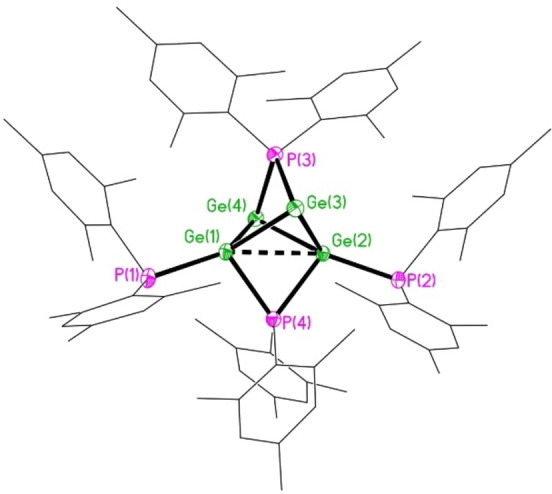
Molecular structure of **7** with 40 % probability ellipsoids and with H atoms and disordered solvent of crystallization omitted for clarity. Selected bond lengths [Å]: Ge(1)⋅⋅⋅Ge(2) 2.9267(3), Ge(1)‐Ge(3) 2.5622(4), Ge(1)‐Ge(4) 2.5802(3), Ge(2)‐Ge(3) 2.5656(3), Ge(2)‐Ge(4) 2.5749(4), Ge(3)⋅⋅⋅Ge(4) 3.5767(1) Ge(1)‐P(1) 2.3617(6), Ge(1)‐P(4) 2.3982(6), Ge(2)‐P(2) 2.3534(6), Ge(2)‐P(4) 2.4088(6), Ge(3)‐P(3) 2.4816(6), Ge(4)‐P(3) 2.4662(6).

Compound **7** crystallizes as a discrete molecular cluster with a unique Ge_4_P_4_ core and an average Ge^I^ oxidation state (Figure 11); the structure contains an additional five molecules of methylcyclohexane which are extensively disordered. The cluster core may be described as a tetrahedron of Ge centers with two non‐adjacent edges bridged by μ_2_‐P(Mes)_2_ ligands, containing a terminal P(Mes)_2_ ligand on two adjacent Ge centers and two three‐coordinate Ge atoms. The four non‐bridged Ge−Ge distances [average 2.5707 Å] are typical for a Ge^I^−Ge^I^ bond, while the two bridged Ge−Ge distances are significantly longer and substantially different to each other [Ge(1)‐Ge(2) 2.9267(3), Ge(3)‐Ge(4) 3.5767(1) Å]. Nonetheless, the longest of these distances is still well within the sum of the van der Waal's radii for two Ge atoms (4.58 Å), suggesting a weak bonding interaction exists between these centers. This is supported by NBO analysis: the WBIs for the non‐bridged Ge−Ge bonds lie in the typical range 0.760–0.810, whereas the two phosphanyl‐bridged Ge⋅⋅⋅Ge interactions have WBIs of 0.117 and 0.063 for the shorter and longer distances, respectively.

The bridging Ge(1)‐P(4) and Ge(2)‐P(4) distances [2.3982(6) and 2.4088(6) Å, respectively] are somewhat longer than the terminal Ge(1)‐P(1) and Ge(2)‐P(2) distances [2.3617(6) and 2.3534(6) Å, respectively], although all of these distances lie within the normal range for Ge−P single bonds.^[11a]^ In contrast, the Ge−P distances to the phosphanyl ligand which bridges the two weakly bonded germanium atoms are rather long [Ge(3)‐P(3) 2.4816(6), Ge(4)‐P(3) 2.4662(6) Å]. The Ge(3)‐P(3)‐Ge(4) angle [92.59(2)°] is correspondingly more obtuse than the Ge(1)‐P(4)‐Ge(2) angle [75.011(18)°].

The solid‐state structure of **7** is consistent with its solution state ^31^P{^1^H} NMR spectrum, indicating that the Ge_4_P_4_ core remains intact in solution. GIAO calculations suggest that the largest resolved P−P coupling constant observed for **7** (485.9 Hz) results from coupling of the distal phosphorus centers P(3) and P(4) [calculated ^3^ *J*(P(3)‐P(4))=432.2 Hz], whereas ^2^ *J*(P(4)‐P(1)/P(2)) is 79.0 Hz [calculated 66.8 Hz]. The remaining coupling constants are too small to be resolved (calculated 4.5 and −7.6 Hz).

**Figure 12:**
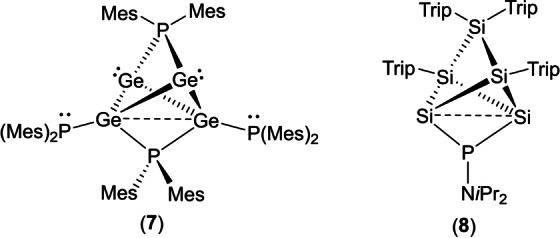
Comparison of the cores of **7** and **8**.

The structure of the Ge_4_P_2_ core of **7** bears some resemblance to the structure of the Si_5_P core of the phosphorus‐functionalized siliconoid Si_5_(Trip)_4_{P(N*i*Pr_2_)} (**8**, Figure 12), which may be described as a tetrahedron of silicon atoms in which non‐adjacent edges are bridged by a Si(Trip)_2_ and a P(N*i*Pr_2_) fragment, but which has an average Si oxidation state of 1.2.^[20]^ Notably, the phosphinidene‐bridged Si⋅⋅⋅Si distance in **8** (2.613(1) Å) is significantly longer than the non‐bridged Si−Si distances (2.391(2) and 2.38(1) Å).

**Scheme 3:**
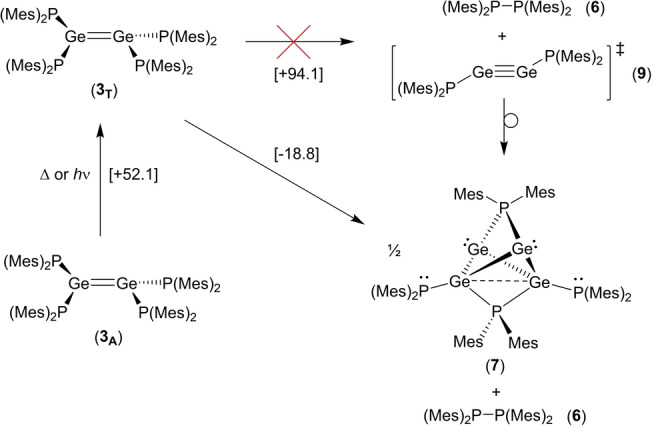
Proposed decomposition of **3** into **7** (free energy changes in square brackets are given in kJ mol^−1^ [(u)B97D/6‐311G(2d,p)]).

The formation of **7** from **3** suggests that, during the decomposition reaction, one equivalent of **6** should be generated for every equivalent of **3** consumed (Scheme 3). Unfortunately, it is not possible to verify this stoichiometry experimentally, since **7** is itself subject to decomposition, generating more diphosphane **6** in the process. However, it is clear from our reaction monitoring experiments that the amount of **6** increases significantly over time as the amount of **3** decreases. To the best of our knowledge, the decomposition of **3** is unique; all previously isolated digermenes are stable towards decomposition (with the exception of their reversion to their germylene monomers).

DFT calculations indicate that the decomposition of **3**, in its singlet ground state (**3_A_**), into **6** and half an equivalent of **7** is mildly endergonic (Δ*G*=+33.3 kJ mol^−1^). However, we calculate that the lowest‐lying excited triplet state (**3_T_**) lies only 52.1 kJ mol^−1^ higher in free energy than **3_A_**. Decomposition of **3_T_** to **6** and half an equivalent of **7** is calculated to be exergonic by −18.8 kJ mol^−1^ (further details of DFT calculations may be found in the Supporting Information).

Triplet state **3_T_** has an optimized geometry which is similar to that of **3_A_**, with a strongly twisted and *trans*‐bent conformation. However, the calculated P⋅⋅⋅P distance in **3_T_** is somewhat shorter than that in **3_A_** (3.480 vs 3.546 Å) and lies well within the sum of the van der Waals radii of two P atoms. In addition, the two proximal phosphorus centers in **3_T_** have calculated spin densities of 0.275 and 0.277. Thus, **3_T_** appears to be appropriately configured for incipient P−P bond formation to generate the diphosphane side‐product **6**.

The foregoing suggests a decomposition process involving thermal or photolytic population of excited triplet state **3_T_**, followed by concerted P−P bond formation/P−Ge bond cleavage and rearrangement to give **6** and the cluster **7**. Although we initially considered that elimination of **6** might lead to the formation of the digermyne {(Mes)_2_P}Ge≡Ge{P(Mes)_2_} (**9**) as an intermediate, before dimerization and rearrangement of the latter to give **7**, DFT calculations indicate that the decomposition of **3_T_** into one equivalent each of **6** and **9** is substantially disfavored (Δ*G*=+94.1 kJ mol^−1^) and so we discount this mechanism.

## Conclusion

The digermene **3** represents the first example of a compound containing a Ge=Ge bond that is substituted by more than two lone pair‐containing heteroatoms. This compound contains one of the most highly distorted element‐element double bonds to have been reported to date, with the structure of **3** being both highly *trans*‐bent *and* significantly twisted in the solid state. This remarkable distortion is associated with π‐stacking interactions between the mesityl rings within the molecules: for the somewhat less *trans*‐bent, non‐twisted, isosteric disilene homologue **2** these interactions lock the conformation of the Si=Si double bond into a non‐twisted configuration, whereas in **3** the π‐stacking interactions permit rotation about the Ge=Ge bond. This is the first time that π‐stacking interactions have been observed to influence the geometric distortion of an element‐element double bond and provides a potentially useful method for controlling E=E distortions in other compounds. ^31^P{^1^H} NMR spectroscopy indicates that this structure is retained in solution at low temperature, the first time NMR spectroscopic evidence for such a twisted structure has been obtained.

Unexpectedly, compound **3** is unstable with respect to formation of the unique Ge^I^ cluster **7** and diphosphane **6**. DFT calculations suggest that this decomposition likely proceeds via excitation of **3** to a low‐lying triplet state, but does not involve formation of the digermyne {(Mes)_2_P}Ge≡Ge{P(Mes)_2_} (**9**) as an intermediate. Overall, the decomposition of **3** appears to be inherently linked to its unusual *trans*‐bent and twisted structure.

## Conflict of interest

The authors declare no financial conflicts of interest.

1

## Supporting information

As a service to our authors and readers, this journal provides supporting information supplied by the authors. Such materials are peer reviewed and may be re‐organized for online delivery, but are not copy‐edited or typeset. Technical support issues arising from supporting information (other than missing files) should be addressed to the authors.

Supporting Information

Supporting Information

Supporting Information

Supporting Information

Supporting Information

## Data Availability

The data that support the findings of this study are available in the Supporting Information of this article and at https://doi.org/10.25405/data.ncl.20079455.
