# Supplementary material for: Spontaneous Decomposition of an Extraordinarily Twisted and Trans‐Bent Fully‐Phosphanyl‐Substituted Digermene to an Unusual GeI Cluster
Source: Angew Chem Int Ed Engl. 2022 Aug 25;61(39):e202208851. doi: 10.1002/anie.202208851 (PMC9804623; doi:10.1002/anie.202208851)

checkCIF/PLATON report

Structure factors have been supplied for datablock(s) kji190019_fa

THIS REPORT IS FOR GUIDANCE ONLY. IF USED AS PART OF A REVIEW PROCEDURE FOR PUBLICATION, IT SHOULD NOT REPLACE THE EXPERTISE OF AN EXPERIENCED CRYSTALLOGRAPHIC REFEREE.

No syntax errors found. CIF dictionary Interpreting this report

Datablock: kji190019_fa

```
Bond precision:  C-C = 0.0041 A          Wavelength=1.54184

Cell:          a=16.0883(4)       b=16.7789(4)    c=20.1691(5)
               alpha=87.4702(19)  beta=68.029(2)  gamma=87.9232(19)
Temperature:  150 K

                Calculated                 Reported
Volume          5043.1(2)                  5043.0(2)
Space group     P -1                       P -1
Hall group      -P 1                       -P 1
Moiety formula  C72 H88 Ge4 P4, 5(C7 H14)  C72 H88 Ge4 P4, 5(C7 H14)
Sum formula     C107 H158 Ge4 P4           C107 H158 Ge4 P4
Mr              1858.66                    1858.56
Dx,g cm-3       1.224                      1.224
Z               2                          2
Mu (mm-1)       2.300                      2.300
F000            1976.0                     1976.0
F000'           1973.20
h,k,lmax        19,20,24                   19,19,24
Nref            17952                      17827
Tmin,Tmax       0.775,0.912                0.724,0.906
Tmin'           0.588

Correction method= # Reported T Limits: Tmin=0.724 Tmax=0.906
AbsCorr = ANALYTICAL

Data completeness= 0.993          Theta(max)= 66.969

R(reflections)= 0.0339( 14546)    wR2(reflections)= 0.0874( 17827)

S = 1.030                   Npar= 1321
```

The following ALERTS were generated. Each ALERT has the format
test-name_ALERT_alert-type_alert-level.
Click on the hyperlinks for more details of the test.

Alert level B

```
PLAT230_ALERT_2_B Hirshfeld Test Diff for    Ge1       --Ge3       .        14.5 s.u.
PLAT230_ALERT_2_B Hirshfeld Test Diff for    Ge2       --Ge3       .        18.4 s.u.
PLAT230_ALERT_2_B Hirshfeld Test Diff for    Ge2       --Ge4       .         9.4 s.u.
PLAT230_ALERT_2_B Hirshfeld Test Diff for    Ge3       --P3        .         7.3 s.u.
```

Alert level C

```
PLAT094_ALERT_2_C Ratio of Maximum / Minimum Residual Density ....        2.55 Report
PLAT220_ALERT_2_C Non-Solvent  Resd 1  C   Ueq(max)/Ueq(min) Range         3.3 Ratio
PLAT230_ALERT_2_C Hirshfeld Test Diff for    Ge1       --Ge4       .         6.3 s.u.
PLAT250_ALERT_2_C Large U3/U1 Ratio for Average U(i,j) Tensor ....         2.3 Note
PLAT250_ALERT_2_C Large U3/U1 Ratio for Average U(i,j) Tensor ....         2.1 Note
PLAT260_ALERT_2_C Large Average Ueq of Residue Including       C87       0.104 Check
PLAT260_ALERT_2_C Large Average Ueq of Residue Including       C94       0.102 Check
PLAT260_ALERT_2_C Large Average Ueq of Residue Including      C101       0.106 Check
PLAT260_ALERT_2_C Large Average Ueq of Residue Including      C87A       0.110 Check
PLAT260_ALERT_2_C Large Average Ueq of Residue Including      C194       0.106 Check
PLAT260_ALERT_2_C Large Average Ueq of Residue Including      C201       0.102 Check
PLAT338_ALERT_4_C Small Aver Tau in Cyclohexane C87A     -C92A           34.43 Degree
PLAT911_ALERT_3_C Missing FCF Refl Between Thmin & STh/L=    0.597         116 Report
```

Alert level G

```
PLAT002_ALERT_2_G Number of Distance or Angle Restraints on AtSite          56 Note
PLAT003_ALERT_2_G Number of Uiso or Uij Restrained non-H Atoms ...         143 Report
PLAT176_ALERT_4_G The CIF-Embedded .res File Contains SADI Records          60 Report
PLAT178_ALERT_4_G The CIF-Embedded .res File Contains SIMU Records           4 Report
PLAT187_ALERT_4_G The CIF-Embedded .res File Contains RIGU Records           1 Report
PLAT300_ALERT_4_G Atom Site Occupancy of C80        Constrained at     0.7005 Check
PLAT300_ALERT_4_G Atom Site Occupancy of C81        Constrained at     0.7005 Check
PLAT300_ALERT_4_G Atom Site Occupancy of C82        Constrained at     0.7005 Check
PLAT300_ALERT_4_G Atom Site Occupancy of C83        Constrained at     0.7005 Check
PLAT300_ALERT_4_G Atom Site Occupancy of C84        Constrained at     0.7005 Check
PLAT300_ALERT_4_G Atom Site Occupancy of C85        Constrained at     0.7005 Check
PLAT300_ALERT_4_G Atom Site Occupancy of C86        Constrained at     0.7005 Check
PLAT300_ALERT_4_G Atom Site Occupancy of H80        Constrained at     0.7005 Check
PLAT300_ALERT_4_G Atom Site Occupancy of H81A       Constrained at     0.7005 Check
PLAT300_ALERT_4_G Atom Site Occupancy of H81B       Constrained at     0.7005 Check
PLAT300_ALERT_4_G Atom Site Occupancy of H82A       Constrained at     0.7005 Check
PLAT300_ALERT_4_G Atom Site Occupancy of H82B       Constrained at     0.7005 Check
PLAT300_ALERT_4_G Atom Site Occupancy of H83A       Constrained at     0.7005 Check
PLAT300_ALERT_4_G Atom Site Occupancy of H83B       Constrained at     0.7005 Check
PLAT300_ALERT_4_G Atom Site Occupancy of H84A       Constrained at     0.7005 Check
PLAT300_ALERT_4_G Atom Site Occupancy of H84B       Constrained at     0.7005 Check
PLAT300_ALERT_4_G Atom Site Occupancy of H85A       Constrained at     0.7005 Check
PLAT300_ALERT_4_G Atom Site Occupancy of H85B       Constrained at     0.7005 Check
PLAT300_ALERT_4_G Atom Site Occupancy of H86A       Constrained at     0.7005 Check
PLAT300_ALERT_4_G Atom Site Occupancy of H86B       Constrained at     0.7005 Check
PLAT300_ALERT_4_G Atom Site Occupancy of H86C       Constrained at     0.7005 Check
PLAT300_ALERT_4_G Atom Site Occupancy of C87        Constrained at     0.8033 Check
PLAT300_ALERT_4_G Atom Site Occupancy of C88        Constrained at     0.8033 Check
PLAT300_ALERT_4_G Atom Site Occupancy of C89        Constrained at     0.8033 Check
PLAT300_ALERT_4_G Atom Site Occupancy of C90        Constrained at     0.8033 Check
PLAT300_ALERT_4_G Atom Site Occupancy of C91        Constrained at     0.8033 Check
PLAT300_ALERT_4_G Atom Site Occupancy of C92        Constrained at     0.8033 Check
PLAT300_ALERT_4_G Atom Site Occupancy of C93        Constrained at     0.8033 Check
PLAT300_ALERT_4_G Atom Site Occupancy of H87        Constrained at     0.8033 Check
PLAT300_ALERT_4_G Atom Site Occupancy of H88A       Constrained at     0.8033 Check
PLAT300_ALERT_4_G Atom Site Occupancy of H88B       Constrained at     0.8033 Check
PLAT300_ALERT_4_G Atom Site Occupancy of H89A       Constrained at     0.8033 Check
```

PLAT300_ALERT_4_G Atom Site Occupancy of H89B Constrained at 0.8033 Check
PLAT300_ALERT_4_G Atom Site Occupancy of H90A Constrained at 0.8033 Check
PLAT300_ALERT_4_G Atom Site Occupancy of H90B Constrained at 0.8033 Check
PLAT300_ALERT_4_G Atom Site Occupancy of H91A Constrained at 0.8033 Check
PLAT300_ALERT_4_G Atom Site Occupancy of H91B Constrained at 0.8033 Check
PLAT300_ALERT_4_G Atom Site Occupancy of H92A Constrained at 0.8033 Check
PLAT300_ALERT_4_G Atom Site Occupancy of H92B Constrained at 0.8033 Check
PLAT300_ALERT_4_G Atom Site Occupancy of H93A Constrained at 0.8033 Check
PLAT300_ALERT_4_G Atom Site Occupancy of H93B Constrained at 0.8033 Check
PLAT300_ALERT_4_G Atom Site Occupancy of H93C Constrained at 0.8033 Check
PLAT300_ALERT_4_G Atom Site Occupancy of C94 Constrained at 0.6001 Check
PLAT300_ALERT_4_G Atom Site Occupancy of C95 Constrained at 0.6001 Check
PLAT300_ALERT_4_G Atom Site Occupancy of C96 Constrained at 0.6001 Check
PLAT300_ALERT_4_G Atom Site Occupancy of C97 Constrained at 0.6001 Check
PLAT300_ALERT_4_G Atom Site Occupancy of C98 Constrained at 0.6001 Check
PLAT300_ALERT_4_G Atom Site Occupancy of C99 Constrained at 0.6001 Check
PLAT300_ALERT_4_G Atom Site Occupancy of C100 Constrained at 0.6001 Check
PLAT300_ALERT_4_G Atom Site Occupancy of H10A Constrained at 0.6001 Check
PLAT300_ALERT_4_G Atom Site Occupancy of H10B Constrained at 0.6001 Check
PLAT300_ALERT_4_G Atom Site Occupancy of H10C Constrained at 0.6001 Check
PLAT300_ALERT_4_G Atom Site Occupancy of H94 Constrained at 0.6001 Check
PLAT300_ALERT_4_G Atom Site Occupancy of H95A Constrained at 0.6001 Check
PLAT300_ALERT_4_G Atom Site Occupancy of H95B Constrained at 0.6001 Check
PLAT300_ALERT_4_G Atom Site Occupancy of H96A Constrained at 0.6001 Check
PLAT300_ALERT_4_G Atom Site Occupancy of H96B Constrained at 0.6001 Check
PLAT300_ALERT_4_G Atom Site Occupancy of H97A Constrained at 0.6001 Check
PLAT300_ALERT_4_G Atom Site Occupancy of H97B Constrained at 0.6001 Check
PLAT300_ALERT_4_G Atom Site Occupancy of H98A Constrained at 0.6001 Check
PLAT300_ALERT_4_G Atom Site Occupancy of H98B Constrained at 0.6001 Check
PLAT300_ALERT_4_G Atom Site Occupancy of H99A Constrained at 0.6001 Check
PLAT300_ALERT_4_G Atom Site Occupancy of H99B Constrained at 0.6001 Check
PLAT300_ALERT_4_G Atom Site Occupancy of C101 Constrained at 0.5216 Check
PLAT300_ALERT_4_G Atom Site Occupancy of C102 Constrained at 0.5216 Check
PLAT300_ALERT_4_G Atom Site Occupancy of C103 Constrained at 0.5216 Check
PLAT300_ALERT_4_G Atom Site Occupancy of C104 Constrained at 0.5216 Check
PLAT300_ALERT_4_G Atom Site Occupancy of C105 Constrained at 0.5216 Check
PLAT300_ALERT_4_G Atom Site Occupancy of C106 Constrained at 0.5216 Check
PLAT300_ALERT_4_G Atom Site Occupancy of C107 Constrained at 0.5216 Check
PLAT300_ALERT_4_G Atom Site Occupancy of H10D Constrained at 0.5216 Check
PLAT300_ALERT_4_G Atom Site Occupancy of H10E Constrained at 0.5216 Check
PLAT300_ALERT_4_G Atom Site Occupancy of H10F Constrained at 0.5216 Check
PLAT300_ALERT_4_G Atom Site Occupancy of H10G Constrained at 0.5216 Check
PLAT300_ALERT_4_G Atom Site Occupancy of H10H Constrained at 0.5216 Check
PLAT300_ALERT_4_G Atom Site Occupancy of H10I Constrained at 0.5216 Check
PLAT300_ALERT_4_G Atom Site Occupancy of H10J Constrained at 0.5216 Check
PLAT300_ALERT_4_G Atom Site Occupancy of H10K Constrained at 0.5216 Check
PLAT300_ALERT_4_G Atom Site Occupancy of H10L Constrained at 0.5216 Check
PLAT300_ALERT_4_G Atom Site Occupancy of H10M Constrained at 0.5216 Check
PLAT300_ALERT_4_G Atom Site Occupancy of H10N Constrained at 0.5216 Check
PLAT300_ALERT_4_G Atom Site Occupancy of H10O Constrained at 0.5216 Check
PLAT300_ALERT_4_G Atom Site Occupancy of H10P Constrained at 0.5216 Check
PLAT300_ALERT_4_G Atom Site Occupancy of H101 Constrained at 0.5216 Check
PLAT300_ALERT_4_G Atom Site Occupancy of C87A Constrained at 0.1967 Check
PLAT300_ALERT_4_G Atom Site Occupancy of C88A Constrained at 0.1967 Check
PLAT300_ALERT_4_G Atom Site Occupancy of C89A Constrained at 0.1967 Check
PLAT300_ALERT_4_G Atom Site Occupancy of C90A Constrained at 0.1967 Check
PLAT300_ALERT_4_G Atom Site Occupancy of C91A Constrained at 0.1967 Check
PLAT300_ALERT_4_G Atom Site Occupancy of C92A Constrained at 0.1967 Check
PLAT300_ALERT_4_G Atom Site Occupancy of C93A Constrained at 0.1967 Check
PLAT300_ALERT_4_G Atom Site Occupancy of H87A Constrained at 0.1967 Check
PLAT300_ALERT_4_G Atom Site Occupancy of H88C Constrained at 0.1967 Check
PLAT300_ALERT_4_G Atom Site Occupancy of H88D Constrained at 0.1967 Check

PLAT300_ALERT_4_G Atom Site Occupancy of H89C Constrained at 0.1967 Check
PLAT300_ALERT_4_G Atom Site Occupancy of H89D Constrained at 0.1967 Check
PLAT300_ALERT_4_G Atom Site Occupancy of H90C Constrained at 0.1967 Check
PLAT300_ALERT_4_G Atom Site Occupancy of H90D Constrained at 0.1967 Check
PLAT300_ALERT_4_G Atom Site Occupancy of H91C Constrained at 0.1967 Check
PLAT300_ALERT_4_G Atom Site Occupancy of H91D Constrained at 0.1967 Check
PLAT300_ALERT_4_G Atom Site Occupancy of H92C Constrained at 0.1967 Check
PLAT300_ALERT_4_G Atom Site Occupancy of H92D Constrained at 0.1967 Check
PLAT300_ALERT_4_G Atom Site Occupancy of H93D Constrained at 0.1967 Check
PLAT300_ALERT_4_G Atom Site Occupancy of H93E Constrained at 0.1967 Check
PLAT300_ALERT_4_G Atom Site Occupancy of H93F Constrained at 0.1967 Check
PLAT300_ALERT_4_G Atom Site Occupancy of C180 Constrained at 0.2995 Check
PLAT300_ALERT_4_G Atom Site Occupancy of C181 Constrained at 0.2995 Check
PLAT300_ALERT_4_G Atom Site Occupancy of C182 Constrained at 0.2995 Check
PLAT300_ALERT_4_G Atom Site Occupancy of C183 Constrained at 0.2995 Check
PLAT300_ALERT_4_G Atom Site Occupancy of C184 Constrained at 0.2995 Check
PLAT300_ALERT_4_G Atom Site Occupancy of C185 Constrained at 0.2995 Check
PLAT300_ALERT_4_G Atom Site Occupancy of C186 Constrained at 0.2995 Check
PLAT300_ALERT_4_G Atom Site Occupancy of H18D Constrained at 0.2995 Check
PLAT300_ALERT_4_G Atom Site Occupancy of H18E Constrained at 0.2995 Check
PLAT300_ALERT_4_G Atom Site Occupancy of H18F Constrained at 0.2995 Check
PLAT300_ALERT_4_G Atom Site Occupancy of H18G Constrained at 0.2995 Check
PLAT300_ALERT_4_G Atom Site Occupancy of H18H Constrained at 0.2995 Check
PLAT300_ALERT_4_G Atom Site Occupancy of H18I Constrained at 0.2995 Check
PLAT300_ALERT_4_G Atom Site Occupancy of H18J Constrained at 0.2995 Check
PLAT300_ALERT_4_G Atom Site Occupancy of H18K Constrained at 0.2995 Check
PLAT300_ALERT_4_G Atom Site Occupancy of H18L Constrained at 0.2995 Check
PLAT300_ALERT_4_G Atom Site Occupancy of H18M Constrained at 0.2995 Check
PLAT300_ALERT_4_G Atom Site Occupancy of H18N Constrained at 0.2995 Check
PLAT300_ALERT_4_G Atom Site Occupancy of H18O Constrained at 0.2995 Check
PLAT300_ALERT_4_G Atom Site Occupancy of H18P Constrained at 0.2995 Check
PLAT300_ALERT_4_G Atom Site Occupancy of H180 Constrained at 0.2995 Check
PLAT300_ALERT_4_G Atom Site Occupancy of C194 Constrained at 0.3999 Check
PLAT300_ALERT_4_G Atom Site Occupancy of C195 Constrained at 0.3999 Check
PLAT300_ALERT_4_G Atom Site Occupancy of C196 Constrained at 0.3999 Check
PLAT300_ALERT_4_G Atom Site Occupancy of C197 Constrained at 0.3999 Check
PLAT300_ALERT_4_G Atom Site Occupancy of C198 Constrained at 0.3999 Check
PLAT300_ALERT_4_G Atom Site Occupancy of C199 Constrained at 0.3999 Check
PLAT300_ALERT_4_G Atom Site Occupancy of C200 Constrained at 0.3999 Check
PLAT300_ALERT_4_G Atom Site Occupancy of H19A Constrained at 0.3999 Check
PLAT300_ALERT_4_G Atom Site Occupancy of H19B Constrained at 0.3999 Check
PLAT300_ALERT_4_G Atom Site Occupancy of H19C Constrained at 0.3999 Check
PLAT300_ALERT_4_G Atom Site Occupancy of H19D Constrained at 0.3999 Check
PLAT300_ALERT_4_G Atom Site Occupancy of H19E Constrained at 0.3999 Check
PLAT300_ALERT_4_G Atom Site Occupancy of H19F Constrained at 0.3999 Check
PLAT300_ALERT_4_G Atom Site Occupancy of H19G Constrained at 0.3999 Check
PLAT300_ALERT_4_G Atom Site Occupancy of H19H Constrained at 0.3999 Check
PLAT300_ALERT_4_G Atom Site Occupancy of H19I Constrained at 0.3999 Check
PLAT300_ALERT_4_G Atom Site Occupancy of H19J Constrained at 0.3999 Check
PLAT300_ALERT_4_G Atom Site Occupancy of H20A Constrained at 0.3999 Check
PLAT300_ALERT_4_G Atom Site Occupancy of H20B Constrained at 0.3999 Check
PLAT300_ALERT_4_G Atom Site Occupancy of H20C Constrained at 0.3999 Check
PLAT300_ALERT_4_G Atom Site Occupancy of H194 Constrained at 0.3999 Check
PLAT300_ALERT_4_G Atom Site Occupancy of C201 Constrained at 0.4784 Check
PLAT300_ALERT_4_G Atom Site Occupancy of C202 Constrained at 0.4784 Check
PLAT300_ALERT_4_G Atom Site Occupancy of C203 Constrained at 0.4784 Check
PLAT300_ALERT_4_G Atom Site Occupancy of C204 Constrained at 0.4784 Check
PLAT300_ALERT_4_G Atom Site Occupancy of C205 Constrained at 0.4784 Check
PLAT300_ALERT_4_G Atom Site Occupancy of C206 Constrained at 0.4784 Check
PLAT300_ALERT_4_G Atom Site Occupancy of C207 Constrained at 0.4784 Check
PLAT300_ALERT_4_G Atom Site Occupancy of H20D Constrained at 0.4784 Check
PLAT300_ALERT_4_G Atom Site Occupancy of H20E Constrained at 0.4784 Check

```
PLAT300_ALERT_4_G Atom Site Occupancy of H20F       Constrained at     0.4784 Check
PLAT300_ALERT_4_G Atom Site Occupancy of H20G       Constrained at     0.4784 Check
PLAT300_ALERT_4_G Atom Site Occupancy of H20H       Constrained at     0.4784 Check
PLAT300_ALERT_4_G Atom Site Occupancy of H20I       Constrained at     0.4784 Check
PLAT300_ALERT_4_G Atom Site Occupancy of H20J       Constrained at     0.4784 Check
PLAT300_ALERT_4_G Atom Site Occupancy of H20K       Constrained at     0.4784 Check
PLAT300_ALERT_4_G Atom Site Occupancy of H20L       Constrained at     0.4784 Check
PLAT300_ALERT_4_G Atom Site Occupancy of H20M       Constrained at     0.4784 Check
PLAT300_ALERT_4_G Atom Site Occupancy of H20N       Constrained at     0.4784 Check
PLAT300_ALERT_4_G Atom Site Occupancy of H20O       Constrained at     0.4784 Check
PLAT300_ALERT_4_G Atom Site Occupancy of H20P       Constrained at     0.4784 Check
PLAT300_ALERT_4_G Atom Site Occupancy of H201       Constrained at     0.4784 Check
PLAT302_ALERT_4_G Anion/Solvent/Minor-Residue Disorder (Resd  3  )       100% Note
PLAT302_ALERT_4_G Anion/Solvent/Minor-Residue Disorder (Resd  4  )       100% Note
PLAT302_ALERT_4_G Anion/Solvent/Minor-Residue Disorder (Resd  5  )       100% Note
PLAT302_ALERT_4_G Anion/Solvent/Minor-Residue Disorder (Resd  6  )       100% Note
PLAT302_ALERT_4_G Anion/Solvent/Minor-Residue Disorder (Resd  7  )       100% Note
PLAT302_ALERT_4_G Anion/Solvent/Minor-Residue Disorder (Resd  8  )       100% Note
PLAT302_ALERT_4_G Anion/Solvent/Minor-Residue Disorder (Resd  9  )       100% Note
PLAT302_ALERT_4_G Anion/Solvent/Minor-Residue Disorder (Resd 10  )       100% Note
PLAT304_ALERT_4_G Non-Integer Number of Atoms in ...... Resd  3         14.71 Check
PLAT304_ALERT_4_G Non-Integer Number of Atoms in ...... Resd  4         16.87 Check
PLAT304_ALERT_4_G Non-Integer Number of Atoms in ...... Resd  5         12.60 Check
PLAT304_ALERT_4_G Non-Integer Number of Atoms in ...... Resd  6         10.95 Check
PLAT304_ALERT_4_G Non-Integer Number of Atoms in ...... Resd  7          4.13 Check
PLAT304_ALERT_4_G Non-Integer Number of Atoms in ...... Resd  8          6.29 Check
PLAT304_ALERT_4_G Non-Integer Number of Atoms in ...... Resd  9          8.40 Check
PLAT304_ALERT_4_G Non-Integer Number of Atoms in ...... Resd 10         10.05 Check
PLAT790_ALERT_4_G Centre of Gravity not Within Unit Cell: Resd.  #          2 Note
              C7 H14
PLAT860_ALERT_3_G Number of Least-Squares Restraints .............       2013 Note
PLAT909_ALERT_3_G Percentage of I>2sig(I) Data at Theta(Max) Still        74% Note
PLAT910_ALERT_3_G Missing # of FCF Reflection(s) Below Theta(Min).          4 Note
PLAT978_ALERT_2_G Number C-C Bonds with Positive Residual Density.          1 Info
```

---

0 **ALERT level A** = Most likely a serious problem - resolve or explain
4 **ALERT level B** = A potentially serious problem, consider carefully
13 **ALERT level C** = Check. Ensure it is not caused by an omission or oversight
194 **ALERT level G** = General information/check it is not something unexpected

0 ALERT type 1 CIF construction/syntax error, inconsistent or missing data
18 ALERT type 2 Indicator that the structure model may be wrong or deficient
4 ALERT type 3 Indicator that the structure quality may be low
189 ALERT type 4 Improvement, methodology, query or suggestion
0 ALERT type 5 Informative message, check

It is advisable to attempt to resolve as many as possible of the alerts in all categories. Often the minor alerts point to easily fixed oversights, errors and omissions in your CIF or refinement strategy, so attention to these fine details can be worthwhile. In order to resolve some of the more serious problems it may be necessary to carry out additional measurements or structure refinements. However, the purpose of your study may justify the reported deviations and the more serious of these should normally be commented upon in the discussion or experimental section of a paper or in the "special_details" fields of the CIF. checkCIF was carefully designed to identify outliers and unusual parameters, but every test has its limitations and alerts that are not important in a particular case may appear. Conversely, the absence of alerts does not guarantee there are no aspects of the results needing attention. It is up to the individual to critically assess their own results and, if necessary, seek expert advice.

Publication of your CIF in IUCr journals

A basic structural check has been run on your CIF. These basic checks will be run on all CIFs submitted for publication in IUCr journals (*Acta Crystallographica*, *Journal of Applied Crystallography*, *Journal of Synchrotron Radiation*); however, if you intend to submit to *Acta Crystallographica Section C* or *E* or *IUCrData*, you should make sure that full publication checks are run on the final version of your CIF prior to submission.

Publication of your CIF in other journals

Please refer to the *Notes for Authors* of the relevant journal for any special instructions relating to CIF submission.

PLATON version of 07/08/2019; check.def file version of 30/07/2019

Datablock kji190019_fa - ellipsoid plot

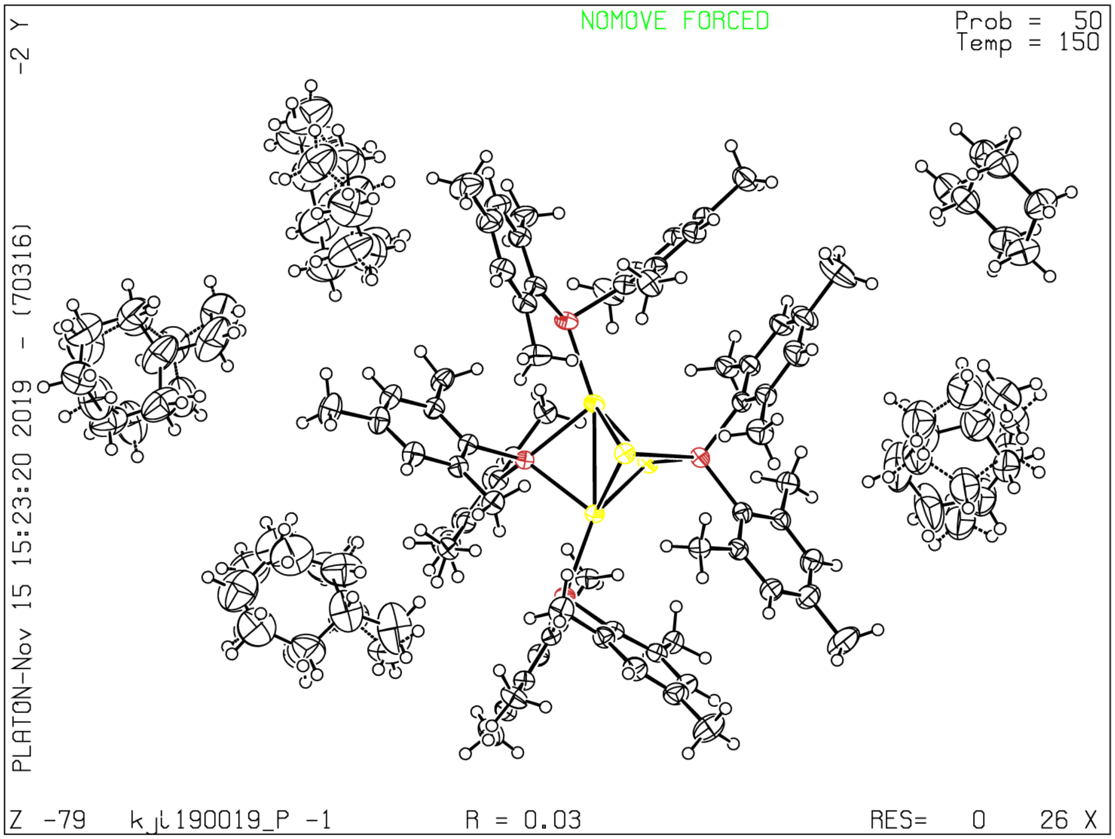

Supplement: Supplementary file 4 — Supporting Information [file ANIE-61-0-s003.pdf]
