# Supplementary material for: Spontaneous Decomposition of an Extraordinarily Twisted and Trans‐Bent Fully‐Phosphanyl‐Substituted Digermene to an Unusual GeI Cluster
Source: Angew Chem Int Ed Engl. 2022 Aug 25;61(39):e202208851. doi: 10.1002/anie.202208851 (PMC9804623; doi:10.1002/anie.202208851)
Supplement: Supplementary file 5 — Supporting Information [file ANIE-61-0-s002.pdf]

Angewandte Chemie
Eine Zeitschrift der Gesellschaft Deutscher Chemiker

Supporting Information

Spontaneous Decomposition of an Extraordinarily Twisted and *Trans*-Bent Fully-Phosphanyl-Substituted Digermene to an Unusual Ge^{I} Cluster

K. Izod, M. Liu, P. Evans, C. Wills, C. M. Dixon, P. G. Waddell, M. R. Probert*

CONTENTS:

S3-S4 General experimental details; details of the syntheses of **3** and **7**; details of the reactions between **3** and 2,3-dimethyl-1,3-butadiene, 1,2-bis(trimethylsilyl)ethyne, and Et_3SiH; X-ray crystallographic details of **3** and **7**.

S5 Details of the calculation of the *trans*-bending and twist angles for **3**.

S5-S6 ^1H, $^{13}C\{^1H\}$, and $^{31}P\{^1H\}$ NMR spectra of **3** in d_8-toluene at 298 K.

S7 $^{31}P\{^1H\}$ EXSY spectrum of **3** in d_8-toluene at 263 K.

S7 Simulation of the $^{31}P\{^1H\}$ NMR spectrum of **3** at 193 K.

S8 $^{31}P\{^1H\}$ CPTOSS-MAS solid-state NMR spectrum of **3** at 298 K.

S8 $^{31}P\{^1H\}$ NMR spectrum of **7** in d_8-toluene at 298 K.

S9 Details of DFT calculations.

S9-S54 Structures, final atomic coordinates, final electronic energies, and number of imaginary frequencies for $\mathbf{3_A}$, $\mathbf{3_B}$, $\mathbf{3_C}$ (both forms), $\mathbf{3_D}$, $\mathbf{3_T}$, **6**, **7**, and **9**.

S52 Alternative views of the HOMO and LUMO of **3**.

S53 **Table S1.** Comparison of key structural parameters (experimental and calculated) for **3**.

S53 **Table S2.** Experimental and calculated chemical shifts (ppm) and coupling constants (Hz) for **7**.

S53 References.

Experimental:

General: All manipulations were carried out using standard Schlenk and dry-box techniques under an atmosphere of dry nitrogen or argon. THF, diethyl ether, toluene, *n*-pentane, and methylcyclohexane were dried prior to use by distillation under nitrogen from sodium, potassium, or sodium/potassium alloy, as appropriate. THF was stored over activated 4A molecular sieves; all other solvents were stored over a potassium film. Deuterated toluene was distilled from potassium under argon and was deoxygenated by three freeze-pump-thaw cycles and stored over activated 4A molecular sieves. $(Mes)_2PH$ was prepared according to a previously published procedure.[S1] *n*-Butyllithium was purchased from Aldrich as a 2.5 M solution in hexanes and its concentration accurately determined by titration before use. All other compounds were used as supplied by the manufacturer.

^{1}H and $^{13}C\{^{1}H\}$ NMR spectra were recorded on a Bruker AvanceIII 500 spectrometer operating at 500.16 and 125.65 MHz, respectively, or a Bruker AvanceIII 300 spectrometer operating at 300.15 and 75.47 MHz, respectively; chemical shifts are quoted in ppm relative to tetramethylsilane. $^{31}P\{^{1}H\}$ solution and solid-state spectra were recorded on a Bruker Avance III HD 500 spectrometer operating at 202.35 MHz; chemical shifts are quoted in ppm relative to external 85% H_3PO_4. The $^{31}P\{^{1}H\}$ EXSY experiment was obtained using a mixing time of 100 ms, with 1024 data points in the t2 dimension and 128 in t1, with subsequent weighting with sine-bell functions and a transformation into a 2K x 2K matrix. The solid-state $^{31}P\{^{1}H\}$ NMR spectra were obtained using cross polarization with total suppression of spinning side bands (CPTOSS), proton decoupling during the acquisition period, a 4 s relaxation delay, 2 ms contact time and at a spin rate of 8 kHz. Adequate signal to noise was achieved with 4096 scans (4.5 h aq. time).

Due to the air- and moisture-sensitive nature of the reported compounds, and to their decomposition at room temperature, satisfactory elemental analyses could not be obtained.

Synthesis of $\{(Mes)_2P\}_2Ge{=}Ge\{P(Mes)_2\}_2$ (3): To a cold (0 °C) solution of $(Mes)_2PH$ (1.14 g, 3.97 mmol)[S1] in Et_2O (20 mL), was added one equivalent of *n*BuLi (1.60 mL of a 2.5 M solution in hexanes). The solution was allowed to warm to room temperature and was stirred for ½ h. This solution was added, dropwise, to a cold (-78 °C) solution of $GeCl_2 \cdot$1,4-dioxane (0.47 g, 2.00 mmol) in diethyl ether (10 mL). The reaction mixture turned deep blue immediately. While maintaining the temperature below -40 °C, the solvent was removed *in vacuo*. The crude solid was extracted into cold (-30 °C) *n*-pentane (20 mL), filtered, the filtrate was concentrated to 4 mL and was stored at -30 °C for 12 h to afford **3** as deep blue, rectangular crystals. The crystals were isolated by filtration and washed with cold (-78 °C) *n*-pentane (5 mL). Yield of crystalline material: 1.05 g, 46%. ^{1}H NMR (d_8-toluene, 298 K): δ 2.03 (s, 3H, *p*-Me), 2.37 (br. s, 6H, *o*-Me), 6.56 (br. s, 2H, ArH). $^{13}C\{^{1}H\}$ NMR (d_8-toluene, 298 K): δ 20.9 (*p*-Me), 24.8 (br., *o*-Me), 129.4 (Ar), 132.9 (d, J_{PC} = 23.4 Hz, Ar), 136.9 (Ar), 143.2 (d, J_{PC} = 11.8 Hz, Ar). $^{31}P\{^{1}H\}$ NMR (d_8-toluene, 298 K): δ -15.1 (br. s).

Attempted reaction between 3 and 2,3-dimethyl-1,3-butadiene: Freshly prepared crystalline **3** (0.06 g, 0.05 mmol) was dissolved in d_8-toluene (0.3 mL) in a Young's valve NMR tube and this solution was cooled to 0 °C. To this solution was added 2,3-dimethyl-1,3-butadiene (0.004 mL, 0.05 mmol). The solution immediately changed from deep blue to pale brown. A $^{31}P\{^{1}H\}$ NMR spectrum of the reaction solution indicated the formation of diphosphane **6** and secondary phosphane **5** as the major products.

Attempted reaction between 3 and 1,2-bis(trimethylsilyl)ethyne: Freshly made crystalline **3** (0.61 g, 0.5 mmol) was dissolved in pentane (10 mL) and to this solution 1,2-bis(trimethylsilyl)ethyne (0.1 mL, 0.5 mmol) was added via syringe. The reaction mixture was stirred at room temperature for 48 h. A $^{31}P\{^{1}H\}$ NMR spectrum of the reaction solution indicated the formation of diphosphane **6** and secondary phosphane **5** as the major products.

Attempted reaction between 3 and triethylsilane: Freshly made crystalline **3** (0.61 g, 0.5 mmol) was dissolved in pentane (10 mL) and to this solution freshly distilled triethylsilane (0.08 mL, 0.5 mmol) was added via syringe. The reaction mixture immediately became pale yellow. A $^{31}P\{^{1}H\}$ NMR spectrum of the reaction solution indicated the formation of diphosphane **6** and secondary phosphane **5** as the only phosphorus-containing products.

Decomposition of 3 into 7: Storage of a solution of **3** in diethyl ether at room temperature for 2 days led to gradual decomposition and formation of a red solution. Concentration and cooling of this solution to 3 °C for 12 h led to deposition of a mixture of colorless crystals of the diphosphane **6** and deep red crystals of **7**. Manual separation of these crystals gave a relatively clean sample of **7**, although this still contained some diphosphane **6** and secondary phosphane **5** and gradually decomposed on standing at room temperature (see main text). $^{31}P\{^{1}H\}$ NMR (d_8-toluene): δ -110.9 (d, J_{PP} = 485.9 Hz), -26.3 (d, J_{PP} = 79.0 Hz), 23.2 (dd, J_{PP} = 485.9, 79.0 Hz).

X-ray crystallography: Data for **3** were collected on a Bruker APEX-II CCD diffractometer, whereas for **7** data were collected on an Xcalibur, Atlas, Gemini ultra diffractometer using an Enhance Ultra X-ray Source ($\lambda_{CuK\alpha}$ = 1.54184 Å). Using an Oxford Cryosystems CryostreamPlus open-flow N_2 cooling device, data for **3** were collected at 200 K, whereas data for **7** were collected at 150 K. Cell refinement, data collection and data reduction were undertaken using CrysAlisPro.[S2] For **7** an analytical numeric absorption correction was applied using a multifaceted crystal model based on expressions derived by R. C. Clark and J. S. Reid.[S3] For **3** intensities were corrected for absorption empirically using spherical harmonics. The structures were solved using XT and refined by XL through the Olex2 interface.[S4,S5] Hydrogen atoms were positioned with idealized geometry and their displacement parameters were constrained using a riding model. The structure of **3** contains large solvent-accessible void space occupied by disordered pentane molecules in multiple orientations. The electron density associated with these solvent molecules is very diffuse and could not be modelled sensibly. It was therefore treated using the Olex2 solvent mask routine. For **7** four of the five methylcyclohexane molecules in the asymmetric unit were modelled as disordered over two positions. The occupancies of the disordered sites were refined independently of the atomic displacement parameters. The geometries of the disordered cyclohexane molecules were restrained using the SADI card and the displacement parameters of all partially-occupied non-hydrogen atoms were restrained using the SIMU card.

Calculation of *trans*-bending and twist angles of 3: The *trans*-bending angle is defined as the angle between the GeP_2 plane and the Ge-Ge vector; this is independent of any twisting of the molecule (Figure S1). This was calculated by positioning a dummy atom X1 midway along the P-P vector and then measuring the X1-Ge-Ge angle (θ).

The twist angle is defined as the deviation of the dihedral angle between normals to the GeP_2 planes from 180°. This is the same as the deviation of the dihedral angle between

X1-Ge-Ge-X2 (φ) from 180°, where X1 and X2 are dummy atoms which are positioned midway along the two respective P-P vectors (Figure S1).

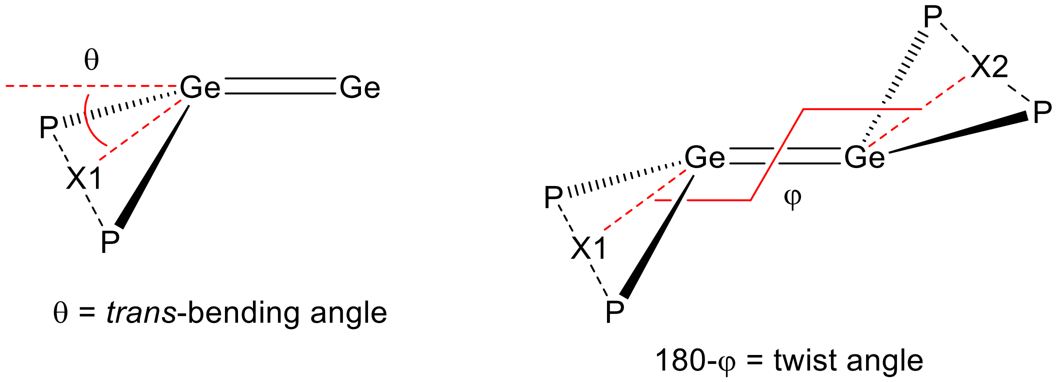

Figure S1. Definition of the *trans*-bending and twist angles in **3**.

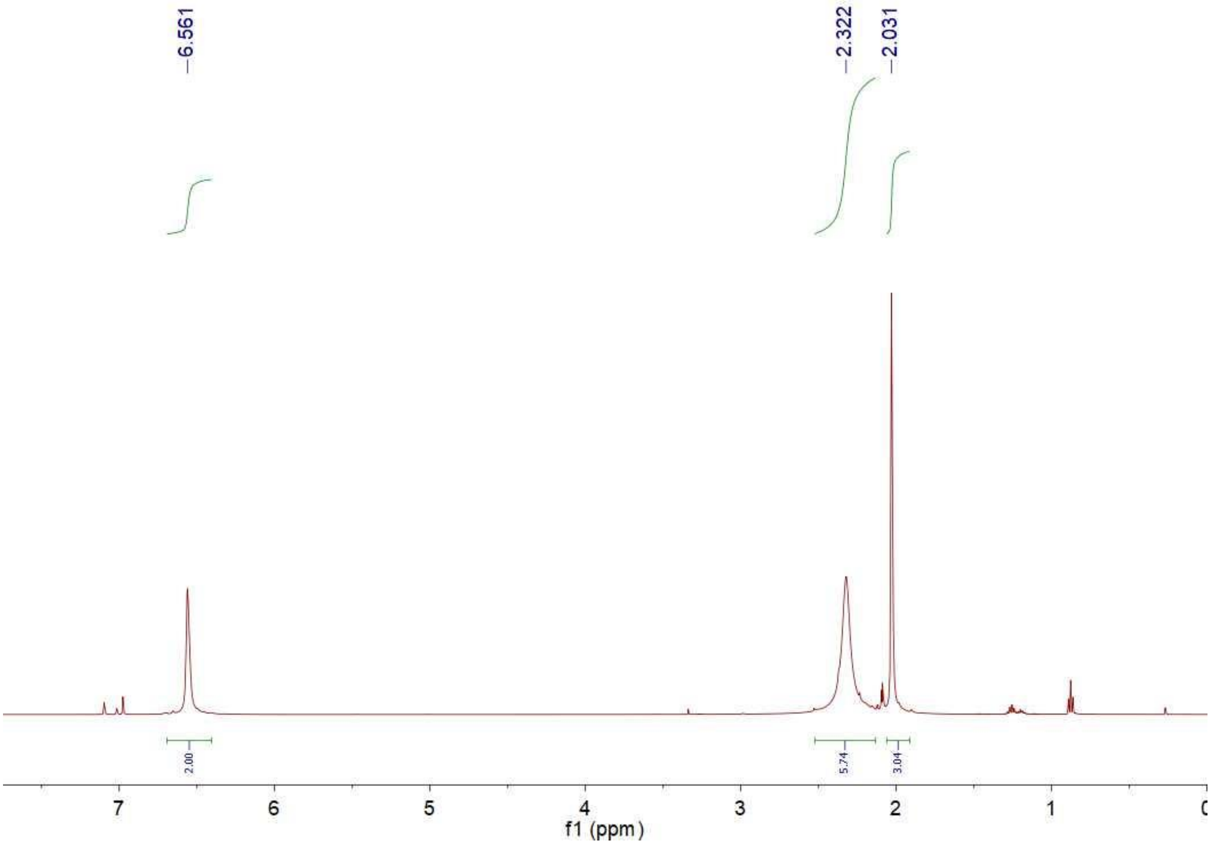

Figure S2. ^{1}H NMR spectrum of **3** in d_8-toluene at 298 K.

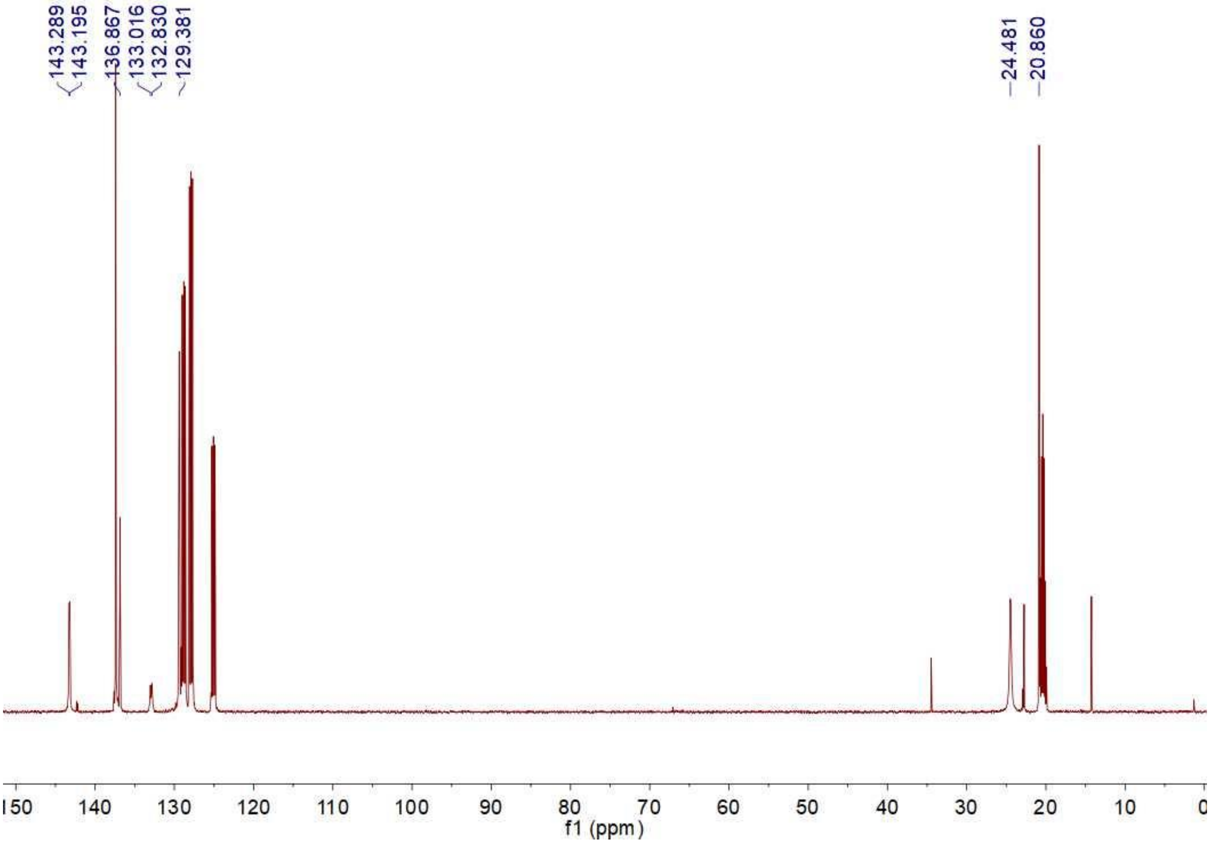

Figure S3. $^{13}C\{^{1}H\}$ NMR spectrum of **3** in d_8-toluene at 298 K.

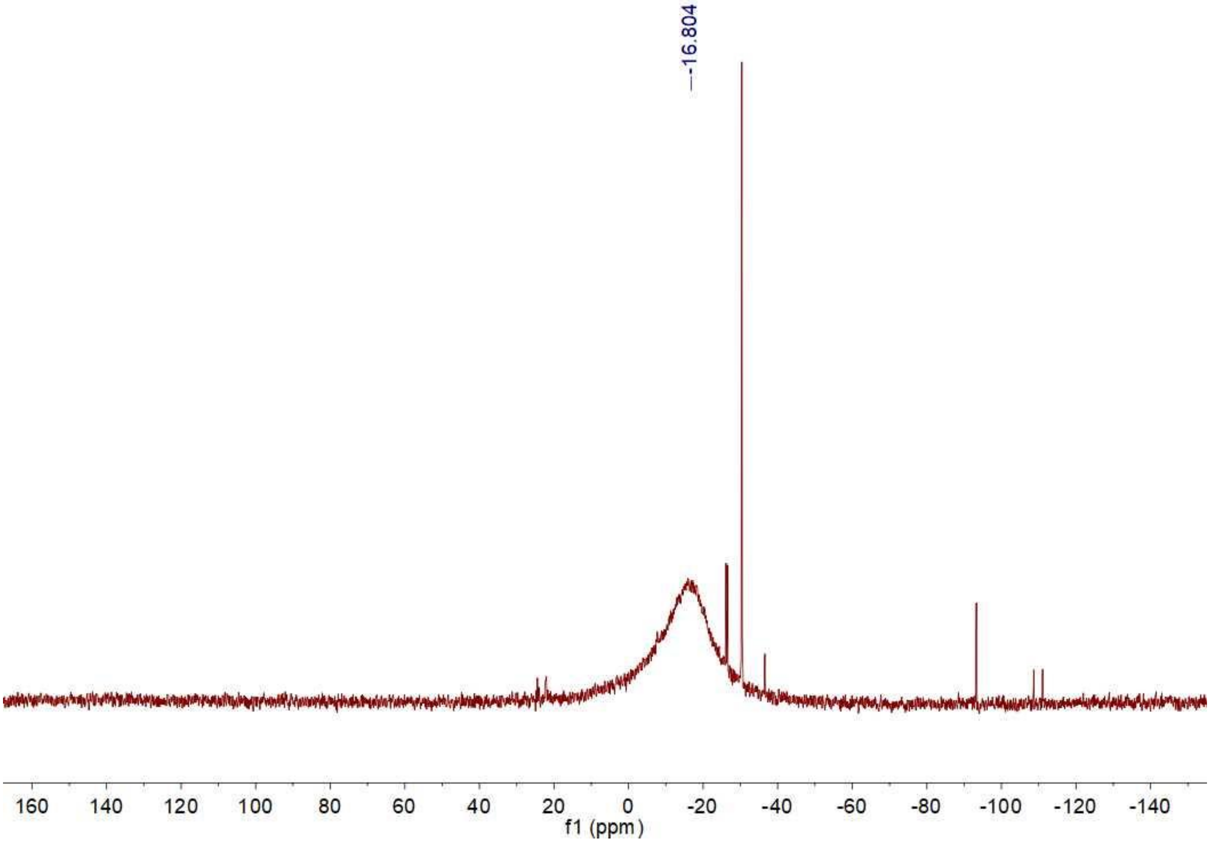

Figure S4. $^{31}P\{^{1}H\}$ NMR spectrum of **3** in d_8-toluene at 298 K.

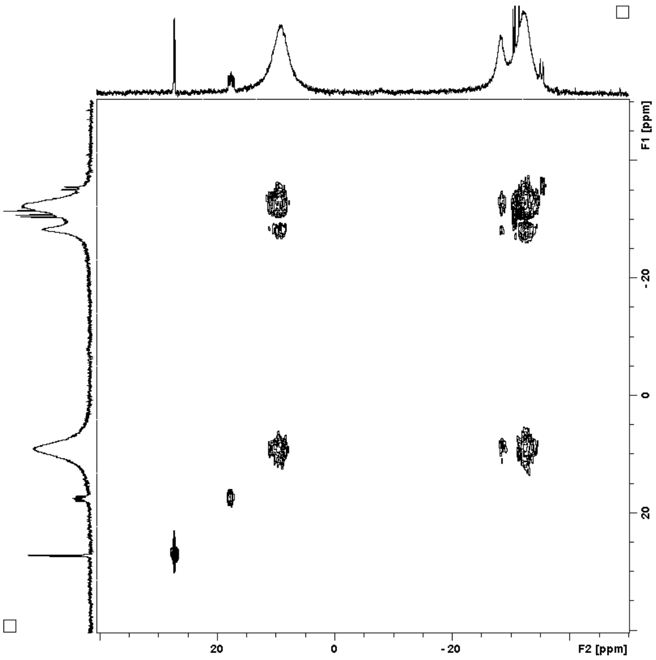

Figure S5. $^{31}P\{^1H\}$ EXSY spectrum of **3** in d_8-toluene at 263 K.

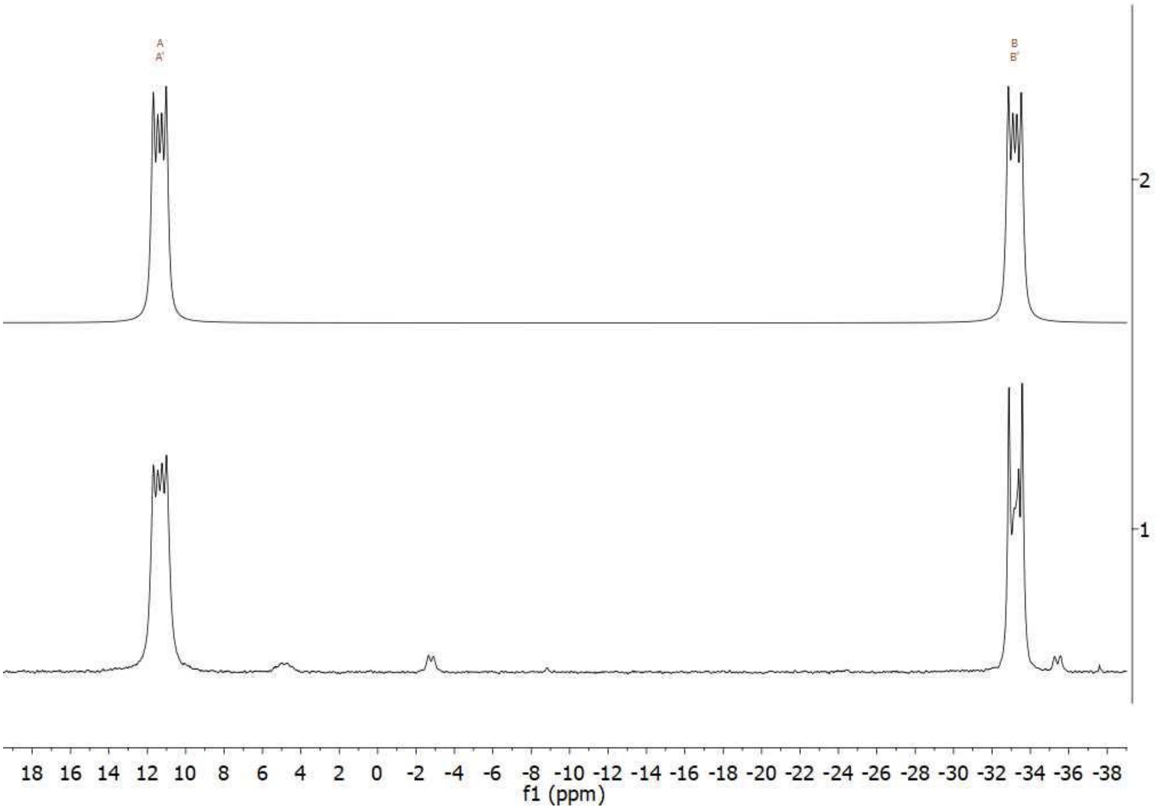

Figure S6. Stacked plot of the simulation (upper) of the 193 K $^{31}P\{^1H\}$ NMR spectrum of **3** in d_8-toluene (lower). The peak at -32.4 ppm is coincident with the peak due to the diphosphane impurity $(Mes)_2P$-$P(Mes)_2$ (**6**) and so the simulation of this peak could not be carried out accurately.

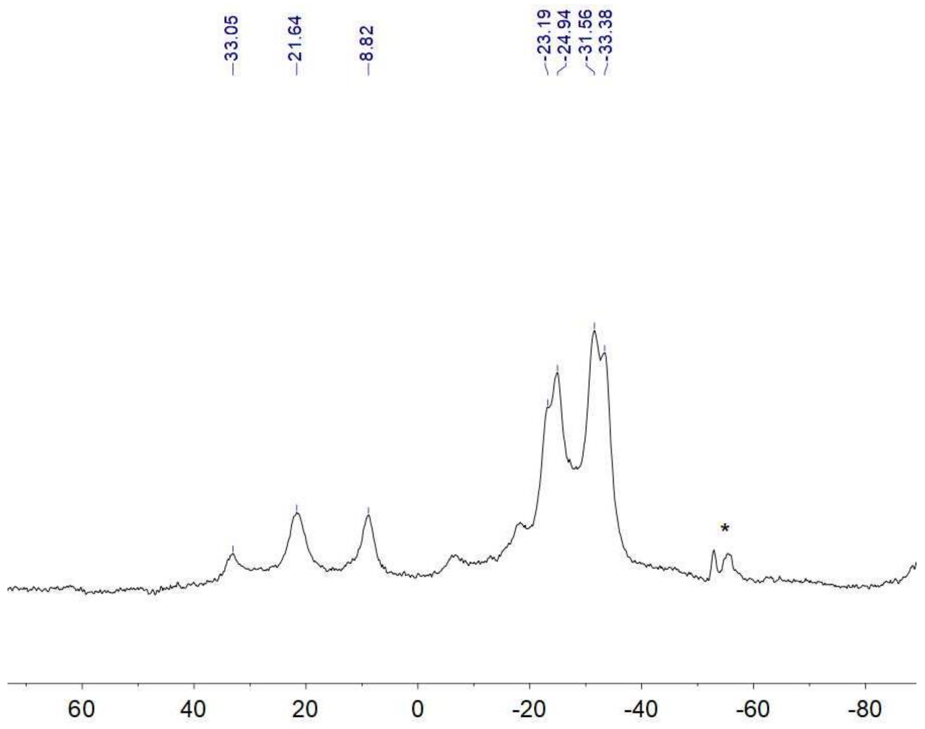

Figure S7. $^{31}P\{^{1}H\}$ CPTOSS-MAS solid-state NMR spectrum of **3** at 298 K (spinning rate 8000 Hz) [* free phosphane **5**].

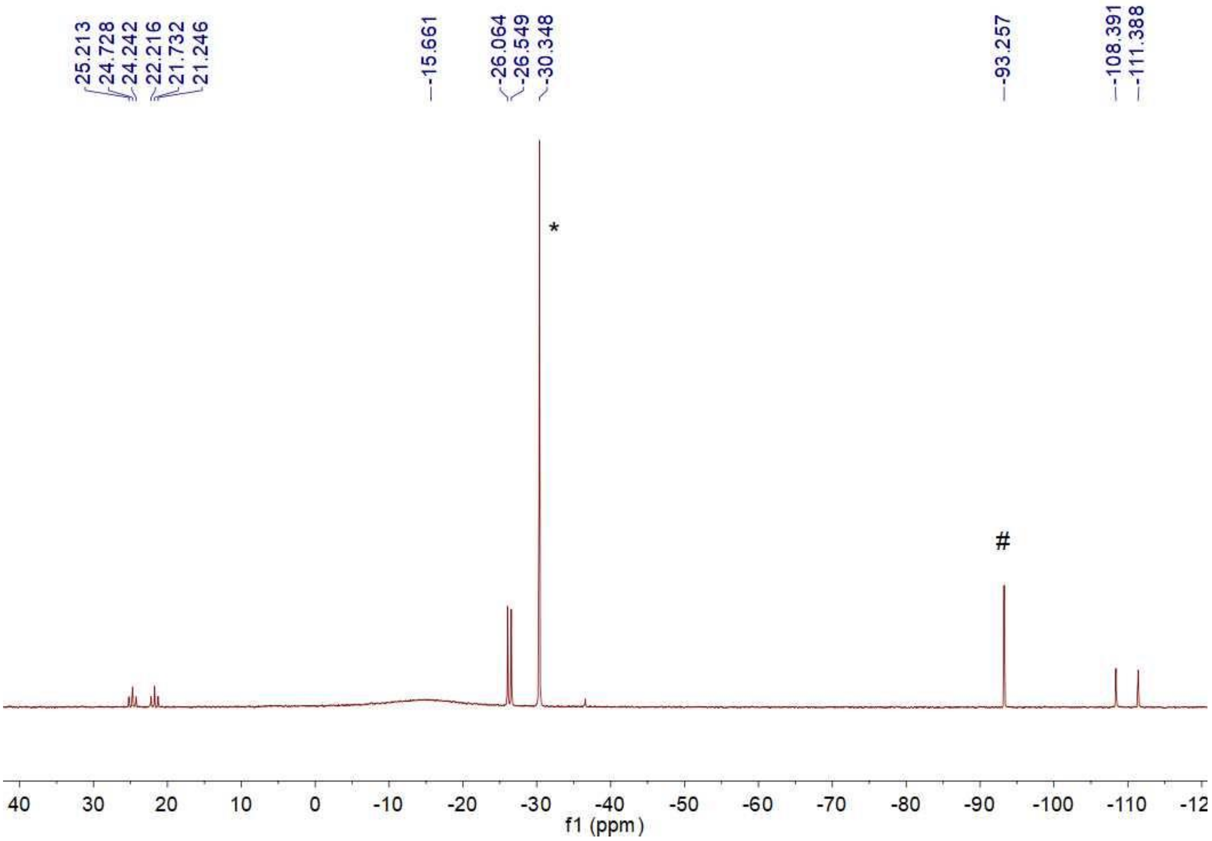

Figure S8. $^{31}P\{^{1}H\}$ NMR spectrum of a sample of **3** in d_8-toluene after 48 hours, showing substantial decomposition to **6** and **7** (labelled peaks due to **6** (*) and **5** (#)).

DFT calculations:

Geometry optimizations were performed with the Gaussian09 suite of programs (revision D.01).[S6]

The B97D functional,[S7] which explicitly includes mid-to-long-range dispersive interactions, generated an optimized geometry for **3** which correlated well with the data obtained by X-ray crystallography (Table S1). In view of this, ground state optimizations and frequency calculations were performed using the B97D functional with the 6-311G(2d,p) all-electron basis set[S8] on all atoms [default parameters were used throughout]. Optimization and frequency calculations on the triplet state $\mathbf{3_T}$ were performed at the unrestricted uB97D/6-311G(2d,p) level of theory. Automatic density fitting was employed for all geometry optimizations and frequency calculations. For the tetrylene $\mathbf{3_C}$ two minimum energy geometries were found, one with two pyramidal P atoms and one with one pyramidal and one planar P atom. These geometries differ in free energy by just 0.1 kJ mol^{-1}; the first of these was the lower in free energy and was used for all further calculations. The global minimum energy conformation of **9** was located by a relaxed potential energy surface scan at the B97D/6-31G* level in which the P-Ge-Ge-P dihedral angle was increased in 10° increments through a 180° rotation; the located minimum energy geometry was then re-optimized at the B97D/6-311G(2d,p) level. The identity of all minima was confirmed by the absence of imaginary vibrational frequencies in each case. Natural Bond Orbital analyses were performed using the NBO 3.1 module of Gaussian09.[S9] NMR shielding tensors and coupling constants were calculated for the optimized structures of **3** and **7** (B97D/6-311G(2d,p)) using the GIAO method at the PBE1PBE/def2QZV [Ge, P], 6-31G(d,p) [C, H] level of theory;[S10] all ^{31}P chemical shifts were calculated relative to PMe_3 at the same level of theory and are quoted relative to 85% H_3PO_4 (see Table S2).

Final atomic coordinates for $\mathbf{3_A}$:

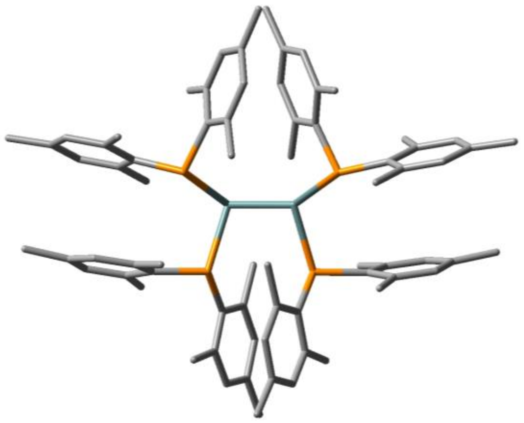

Center	Atomic	Atomic	Coordinates (Angstroms)		
Number	Number	Type	X	Y	Z

1	32	0	-1.094728	0.279821	-0.633727
2	32	0	1.095432	0.270696	0.640834
3	15	0	-2.577864	1.374472	0.846016
4	15	0	-1.781188	-1.949343	-0.130951
5	15	0	1.754286	-1.966004	0.137750
6	15	0	2.594714	1.343943	-0.837708
7	6	0	-1.766611	2.995626	1.229397
8	6	0	-1.819790	4.135993	0.388626
9	6	0	-1.281383	5.345279	0.840888
10	6	0	-0.653303	5.464741	2.082020
11	6	0	-0.565738	4.322645	2.882212
12	6	0	-1.116069	3.097422	2.489498
13	6	0	-2.369741	4.082608	-1.014697
14	6	0	-0.077168	6.785220	2.536068
15	6	0	-0.984593	1.917789	3.427856
16	6	0	-4.258111	1.775134	0.175610
17	6	0	-4.721162	1.545493	-1.145357
18	6	0	-6.064427	1.795576	-1.455085
19	6	0	-6.975521	2.274822	-0.512366
20	6	0	-6.504394	2.513017	0.780523
21	6	0	-5.175491	2.267937	1.145551
22	6	0	-3.833459	1.062471	-2.264619
23	6	0	-8.425881	2.496989	-0.874026
24	6	0	-4.773591	2.535205	2.581346
25	6	0	-3.623367	-1.941597	-0.023767
26	6	0	-4.489953	-2.077286	-1.138843
27	6	0	-5.862157	-1.858315	-0.964141
28	6	0	-6.414230	-1.532465	0.276489
29	6	0	-5.560951	-1.505468	1.382938
30	6	0	-4.184512	-1.719019	1.265000

31	6	0	-4.001978	-2.458308	-2.518895
32	6	0	-7.878842	-1.199477	0.425480
33	6	0	-3.347362	-1.720416	2.526915
34	6	0	-1.279731	-3.278263	-1.307553
35	6	0	-0.589361	-3.094277	-2.533957
36	6	0	-0.203048	-4.215766	-3.280161
37	6	0	-0.470068	-5.521374	-2.862508
38	6	0	-1.146799	-5.688659	-1.652668
39	6	0	-1.550134	-4.604036	-0.866276
40	6	0	-0.249894	-1.738409	-3.097335
41	6	0	-0.045368	-6.709389	-3.694189
42	6	0	-2.241540	-4.895931	0.446912
43	6	0	1.227962	-3.289667	1.308855
44	6	0	1.477610	-4.618769	0.864618
45	6	0	1.054897	-5.698514	1.647237
46	6	0	0.375977	-5.522944	2.854939
47	6	0	0.136561	-4.214348	3.279318
48	6	0	0.541834	-3.097284	2.536218
49	6	0	2.169914	-4.918393	-0.446324
50	6	0	-0.125972	-6.705221	3.649940
51	6	0	0.229706	-1.737682	3.106204
52	6	0	3.595951	-1.986777	0.030018
53	6	0	4.160169	-1.771838	-1.258657
54	6	0	5.539502	-1.578042	-1.376292
55	6	0	6.392147	-1.617449	-0.269712
56	6	0	5.835421	-1.935853	0.970754
57	6	0	4.460233	-2.135639	1.145100
58	6	0	3.323419	-1.761487	-2.520805
59	6	0	7.861501	-1.306107	-0.418954
60	6	0	3.966257	-2.511584	2.524346
61	6	0	4.281409	1.714845	-0.168663

62	6	0	5.208091	2.187975	-1.139473
63	6	0	6.540214	2.413424	-0.773533
64	6	0	7.005296	2.174471	0.521381
65	6	0	6.085160	1.714133	1.464814
66	6	0	4.738735	1.483420	1.153975
67	6	0	4.812563	2.455726	-2.576938
68	6	0	8.458277	2.375551	0.884692
69	6	0	3.842737	1.016804	2.273635
70	6	0	1.816761	2.978335	-1.232138
71	6	0	1.167208	3.082873	-2.492894
72	6	0	0.649413	4.317748	-2.899388
73	6	0	0.767604	5.466041	-2.112128
74	6	0	1.403033	5.346292	-0.874796
75	6	0	1.906893	4.127997	-0.407217
76	6	0	1.009185	1.898053	-3.420460
77	6	0	0.170645	6.781674	-2.551338
78	6	0	2.468832	4.081101	0.991548
79	1	0	-8.902373	3.208239	-0.187334
80	1	0	-8.990699	1.553448	-0.821324
81	1	0	-8.525670	2.880692	-1.898056
82	1	0	-2.962613	1.716742	-2.397578
83	1	0	-4.387007	1.030635	-3.211069
84	1	0	-3.463900	0.053469	-2.065440
85	1	0	-4.352420	1.630521	3.043150
86	1	0	-5.642903	2.854976	3.169016
87	1	0	-4.002502	3.314115	2.648950
88	1	0	-7.191527	2.895216	1.536462
89	1	0	-6.404724	1.613512	-2.475079
90	1	0	-3.105300	-1.899275	-2.806389
91	1	0	-4.784919	-2.273343	-3.264622
92	1	0	-3.724938	-3.520267	-2.559034

93	1	0	-8.321654	-1.719656	1.286234
94	1	0	-8.444029	-1.472021	-0.474824
95	1	0	-8.001349	-0.119574	0.591917
96	1	0	-2.689135	-2.597921	2.563667
97	1	0	-3.997439	-1.726129	3.411470
98	1	0	-2.710238	-0.827810	2.579081
99	1	0	-5.975862	-1.307124	2.371797
100	1	0	-6.516084	-1.925652	-1.834160
101	1	0	0.181920	-1.831645	-4.101257
102	1	0	-1.137975	-1.093792	-3.164357
103	1	0	0.482576	-1.226254	-2.462812
104	1	0	0.331622	-4.056635	-4.217205
105	1	0	0.907014	-6.515006	-4.204809
106	1	0	-0.793567	-6.937096	-4.468969
107	1	0	0.070174	-7.606274	-3.072018
108	1	0	-1.351611	-6.697147	-1.291903
109	1	0	-1.707673	-4.418062	1.278917
110	1	0	-2.269986	-5.976739	0.630308
111	1	0	-3.271236	-4.513563	0.455855
112	1	0	2.183687	-5.998960	-0.632396
113	1	0	1.644139	-4.432655	-1.279021
114	1	0	3.204600	-4.549771	-0.452117
115	1	0	1.250099	-6.709185	1.287214
116	1	0	0.499645	-7.591539	3.482887
117	1	0	-0.139576	-6.484666	4.725347
118	1	0	-1.154370	-6.964901	3.354402
119	1	0	-0.387603	-4.049457	4.221290
120	1	0	1.128338	-1.107592	3.169866
121	1	0	-0.498134	-1.211361	2.477739
122	1	0	-0.197690	-1.826802	4.112423
123	1	0	4.753532	-2.344939	3.269817

124	1	0	3.081608	-1.935350	2.815177
125	1	0	3.667831	-3.567879	2.561400
126	1	0	6.488266	-2.012975	1.840766
127	1	0	7.999401	-0.229110	-0.591731
128	1	0	8.297800	-1.837668	-1.276104
129	1	0	8.421858	-1.581396	0.483508
130	1	0	5.957412	-1.385415	-2.365021
131	1	0	3.973833	-1.774003	-3.405055
132	1	0	2.654971	-2.631155	-2.558821
133	1	0	2.696707	-0.861527	-2.572592
134	1	0	5.688544	2.754381	-3.165799
135	1	0	4.372883	1.557559	-3.034027
136	1	0	4.057754	3.250001	-2.649164
137	1	0	7.234459	2.780811	-1.530300
138	1	0	8.562153	2.764856	1.906202
139	1	0	8.947835	3.073993	0.194055
140	1	0	9.007687	1.422566	0.839978
141	1	0	6.420998	1.531626	2.486214
142	1	0	4.395144	0.978276	3.220486
143	1	0	2.981236	1.683889	2.405343
144	1	0	3.458210	0.013005	2.076041
145	1	0	1.983643	1.488240	-3.717502
146	1	0	0.454115	1.083360	-2.931462
147	1	0	0.459828	2.188625	-4.324877
148	1	0	0.146638	4.382466	-3.865132
149	1	0	0.076741	6.834018	-3.643464
150	1	0	-0.837549	6.911868	-2.126388
151	1	0	0.779602	7.629653	-2.211362
152	1	0	1.483134	6.221516	-0.230524
153	1	0	2.304441	5.036292	1.502583
154	1	0	1.955495	3.300926	1.566607

155	1	0	3.541004	3.853257	1.003490
156	1	0	-1.857960	3.294391	-1.580224
157	1	0	-2.194544	5.032232	-1.532472
158	1	0	-3.443188	3.862682	-1.033667
159	1	0	-1.321491	6.210406	0.179748
160	1	0	0.741750	6.636141	3.251591
161	1	0	-0.842247	7.402743	3.031081
162	1	0	0.306172	7.361851	1.683660
163	1	0	-0.056032	4.383510	3.844530
164	1	0	-0.430701	2.204739	4.330648
165	1	0	-1.968066	1.531735	3.726826
166	1	0	-0.446243	1.087331	2.947063

Final electronic energy: -8317.02132574 a.u.

NIMAG = 0

Final atomic coordinates for $\mathbf{3_B}$:

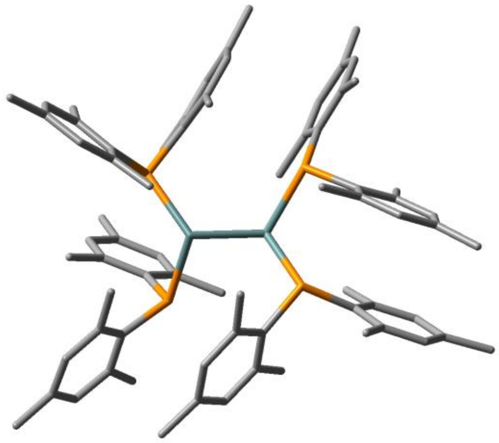

Center Number	Atomic Number	Atomic Type	Coordinates (Angstroms) X	Y	Z
1	15	0	2.223588	-1.549681	1.040414
2	15	0	2.231946	2.068728	-0.375053

3	6	0	3.795020	-2.437911	0.599977
4	6	0	4.343424	-3.222091	1.653879
5	6	0	5.514826	-3.958947	1.431944
6	1	0	5.924841	-4.543841	2.256375
7	6	0	6.161004	-3.980601	0.194768
8	6	0	5.587896	-3.240882	-0.842524
9	1	0	6.062009	-3.247530	-1.824851
10	6	0	4.426629	-2.478963	-0.667276
11	6	0	3.710956	-3.311798	3.028041
12	1	0	3.798955	-2.367767	3.581203
13	1	0	4.190078	-4.104583	3.615810
14	1	0	2.636554	-3.532917	2.950164
15	6	0	7.400178	-4.815234	-0.032334
16	1	0	7.142814	-5.787867	-0.479737
17	1	0	7.924350	-5.012402	0.911502
18	1	0	8.095792	-4.313842	-0.718401
19	6	0	3.914126	-1.711527	-1.857846
20	1	0	2.877986	-1.987075	-2.090925
21	1	0	4.526190	-1.913799	-2.745798
22	1	0	3.942651	-0.627472	-1.678299
23	6	0	2.759722	-0.339383	2.340182
24	6	0	1.899106	-0.192379	3.464604
25	6	0	2.247316	0.689433	4.494592
26	1	0	1.573085	0.794549	5.345545
27	6	0	3.427329	1.435240	4.461297
28	6	0	4.269677	1.274002	3.357502
29	1	0	5.194231	1.851128	3.307597
30	6	0	3.964634	0.411409	2.298020
31	6	0	0.606217	-0.961862	3.590674
32	1	0	-0.094550	-0.687252	2.786647
33	1	0	0.119803	-0.753082	4.551893

34	1	0	0.772045	-2.042190	3.497798
35	6	0	3.759076	2.422402	5.555629
36	1	0	3.391226	3.427377	5.296362
37	1	0	4.843516	2.499264	5.708351
38	1	0	3.292040	2.134420	6.506407
39	6	0	4.950269	0.325717	1.157159
40	1	0	5.573670	-0.575322	1.231743
41	1	0	5.606879	1.204281	1.155608
42	1	0	4.434742	0.281162	0.193395
43	6	0	3.164537	2.588950	-1.904051
44	6	0	3.173650	1.978726	-3.188033
45	6	0	4.073255	2.441784	-4.163115
46	1	0	4.064817	1.965162	-5.144311
47	6	0	4.952091	3.499656	-3.937062
48	6	0	4.906177	4.120626	-2.685631
49	1	0	5.564663	4.966915	-2.485590
50	6	0	4.046247	3.689183	-1.671786
51	6	0	2.230717	0.877623	-3.623247
52	1	0	2.546824	-0.108099	-3.266161
53	1	0	2.191313	0.830118	-4.719274
54	1	0	1.215731	1.045042	-3.254145
55	6	0	5.923928	3.958075	-4.999965
56	1	0	6.937927	3.582705	-4.793650
57	1	0	5.983935	5.054299	-5.036277
58	1	0	5.628252	3.593003	-5.991765
59	6	0	4.087391	4.435729	-0.352857
60	1	0	3.124244	4.914488	-0.131002
61	1	0	4.862402	5.211855	-0.378449
62	1	0	4.298117	3.752726	0.482168
63	6	0	0.909336	3.360975	-0.153284
64	6	0	0.818848	3.914657	1.154428

65	6	0	-0.025232	5.006718	1.384916
66	1	0	-0.087017	5.415339	2.393979
67	6	0	-0.786799	5.585657	0.368573
68	6	0	-0.721811	5.007766	-0.900557
69	1	0	-1.340378	5.414405	-1.701026
70	6	0	0.099302	3.909199	-1.183145
71	6	0	1.610550	3.368537	2.322090
72	1	0	1.483608	2.287288	2.441126
73	1	0	1.295320	3.853092	3.254897
74	1	0	2.687027	3.533743	2.188926
75	6	0	-1.645834	6.798712	0.629156
76	1	0	-1.124717	7.719906	0.325547
77	1	0	-1.890285	6.884457	1.694539
78	1	0	-2.585099	6.740808	0.065937
79	6	0	0.043305	3.329513	-2.573480
80	1	0	1.020430	3.352712	-3.066775
81	1	0	-0.672587	3.886095	-3.190933
82	1	0	-0.297762	2.286670	-2.530852
83	15	0	-2.465836	1.330121	-0.785730
84	15	0	-1.780271	-2.239348	-0.698811
85	6	0	-3.065796	2.688299	0.316014
86	6	0	-3.975674	3.588175	-0.309295
87	6	0	-4.509933	4.649417	0.427032
88	1	0	-5.215243	5.320674	-0.064850
89	6	0	-4.161288	4.880329	1.760373
90	6	0	-3.238793	4.013701	2.344827
91	1	0	-2.937071	4.182356	3.378921
92	6	0	-2.681424	2.927353	1.656894
93	6	0	-4.404270	3.445956	-1.754934
94	1	0	-5.021369	2.550755	-1.909029
95	1	0	-4.982141	4.323839	-2.068952

96	1	0	-3.531850	3.346082	-2.416867
97	6	0	-4.726686	6.057743	2.518132
98	1	0	-4.238791	6.993028	2.204108
99	1	0	-5.802946	6.171971	2.330666
100	1	0	-4.572300	5.946008	3.598794
101	6	0	-1.716551	2.052876	2.418118
102	1	0	-0.752046	1.970570	1.906582
103	1	0	-1.525262	2.467787	3.414943
104	1	0	-2.116539	1.036500	2.548985
105	6	0	-3.950566	0.291917	-1.174920
106	6	0	-3.978394	-0.282604	-2.474887
107	6	0	-5.037000	-1.123927	-2.831133
108	1	0	-5.041362	-1.566677	-3.827834
109	6	0	-6.082589	-1.414677	-1.950422
110	6	0	-6.053937	-0.823200	-0.686694
111	1	0	-6.852808	-1.044047	0.021023
112	6	0	-5.012619	0.017689	-0.276532
113	6	0	-2.884510	-0.031265	-3.490137
114	1	0	-1.922242	-0.419694	-3.129576
115	1	0	-3.120552	-0.530830	-4.438075
116	1	0	-2.752753	1.042890	-3.679533
117	6	0	-7.185773	-2.370393	-2.335516
118	1	0	-6.950627	-3.383481	-1.977052
119	1	0	-8.143114	-2.075342	-1.885862
120	1	0	-7.311069	-2.418172	-3.424916
121	6	0	-5.081787	0.584431	1.122115
122	1	0	-5.430018	1.626022	1.117329
123	1	0	-5.770187	-0.010713	1.734239
124	1	0	-4.099468	0.587326	1.600089
125	6	0	-3.244637	-2.988503	0.152838
126	6	0	-3.780659	-2.663865	1.423368

127	6	0	-5.012369	-3.216281	1.809953
128	1	0	-5.420744	-2.941090	2.783492
129	6	0	-5.721752	-4.106229	1.007430
130	6	0	-5.152691	-4.467189	-0.218047
131	1	0	-5.670910	-5.182683	-0.857605
132	6	0	-3.949405	-3.919408	-0.666611
133	6	0	-3.099675	-1.814531	2.472045
134	1	0	-3.823270	-1.154024	2.962596
135	1	0	-2.656485	-2.458552	3.244730
136	1	0	-2.295129	-1.197260	2.067508
137	6	0	-7.068003	-4.646921	1.428013
138	1	0	-7.879733	-4.129053	0.894320
139	1	0	-7.157176	-5.716781	1.195982
140	1	0	-7.233585	-4.508906	2.503871
141	6	0	-3.442339	-4.341154	-2.030519
142	1	0	-2.426349	-4.755584	-1.976986
143	1	0	-4.103295	-5.102197	-2.463731
144	1	0	-3.409265	-3.479106	-2.711071
145	6	0	-0.369743	-3.433596	-0.624016
146	6	0	0.405803	-3.552768	-1.809055
147	6	0	1.518572	-4.401854	-1.818532
148	1	0	2.118646	-4.469523	-2.726295
149	6	0	1.885136	-5.158837	-0.703077
150	6	0	1.091340	-5.057026	0.442003
151	1	0	1.363850	-5.630202	1.328683
152	6	0	-0.015119	-4.204891	0.510587
153	6	0	0.092741	-2.770400	-3.066858
154	1	0	0.156132	-1.687193	-2.882321
155	1	0	0.806676	-3.022108	-3.861030
156	1	0	-0.922996	-2.978351	-3.427150
157	6	0	3.127121	-6.014975	-0.708930

158	1	0	2.963236	-6.955274	-0.165450
159	1	0	3.442525	-6.251713	-1.733076
160	1	0	3.951954	-5.481559	-0.216252
161	6	0	-0.744220	-4.084754	1.823698
162	1	0	-1.774945	-4.453924	1.757075
163	1	0	-0.219901	-4.645807	2.606982
164	1	0	-0.794303	-3.034095	2.127400
165	32	0	-0.991800	-0.231132	0.187636
166	32	0	1.272434	-0.094843	-0.638880

Final electronic energy: -8317.00896012 a.u.

NIMAG = 0

Final atomic coordinates for $\mathbf{3_C}$ (one planar and one pyramidal P atom):

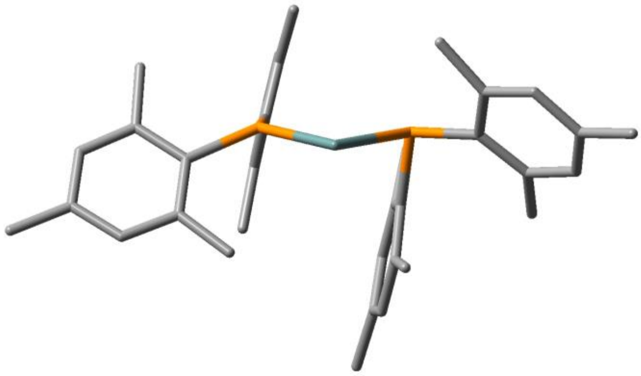

Center Number	Atomic Number	Atomic Type	Coordinates (Angstroms) X	Y	Z
1	32	0	0.591435	1.843667	-0.215552
2	15	0	-1.409674	0.968766	-0.796538
3	15	0	1.829546	-0.201398	-0.714801
4	6	0	-2.941183	1.643304	-0.043306
5	6	0	-4.134097	1.603803	-0.817363
6	6	0	-5.332352	2.055253	-0.253714

7	1	0	-6.240538	2.015138	-0.855956
8	6	0	-5.397395	2.561091	1.047123
9	6	0	-4.215391	2.601750	1.791734
10	1	0	-4.246398	2.982839	2.813020
11	6	0	-2.989140	2.157620	1.280508
12	6	0	-1.784940	2.235168	2.193333
13	1	0	-1.198321	1.308298	2.159701
14	6	0	-4.162969	1.087159	-2.238723
15	1	0	-4.015458	-0.000087	-2.274119
16	6	0	-1.727069	-0.791016	-1.209317
17	6	0	-2.218700	-1.690954	-0.231974
18	6	0	-2.526249	-3.002221	-0.614889
19	1	0	-2.898272	-3.692521	0.142784
20	6	0	-2.356297	-3.453740	-1.925886
21	6	0	-1.849470	-2.555865	-2.871769
22	1	0	-1.693735	-2.892385	-3.897186
23	6	0	-1.521027	-1.236791	-2.540565
24	6	0	-2.393653	-1.293895	1.213111
25	1	0	-3.214352	-0.576092	1.337522
26	6	0	-0.932063	-0.343951	-3.608777
27	1	0	0.102613	-0.077094	-3.352632
28	6	0	1.351825	-1.133389	0.838388
29	6	0	0.889171	-2.463593	0.654935
30	6	0	0.382418	-3.176609	1.747922
31	1	0	0.023548	-4.193255	1.584842
32	6	0	0.324843	-2.629368	3.030988
33	6	0	0.841571	-1.344090	3.211507
34	1	0	0.855455	-0.911815	4.213220
35	6	0	1.358953	-0.591408	2.148982
36	6	0	1.950154	0.761460	2.497671
37	1	0	2.673046	1.104044	1.751430

38	6	0	0.967621	-3.173145	-0.679196
39	1	0	1.999268	-3.181212	-1.058883
40	6	0	3.586250	0.395789	-0.521460
41	6	0	4.003699	1.468091	-1.362883
42	6	0	5.302585	1.975370	-1.252197
43	1	0	5.595836	2.798608	-1.905654
44	6	0	6.235847	1.448271	-0.356064
45	6	0	5.847996	0.331884	0.386129
46	1	0	6.579683	-0.152539	1.034632
47	6	0	4.561306	-0.221523	0.309971
48	6	0	4.326857	-1.507108	1.080016
49	1	0	3.791672	-1.345734	2.023626
50	6	0	3.120185	2.061075	-2.446810
51	1	0	2.537051	1.286844	-2.964171
52	1	0	-2.102180	2.414216	3.228303
53	1	0	-1.112879	3.053529	1.900484
54	1	0	-5.122867	1.323430	-2.713321
55	1	0	-3.356926	1.535673	-2.835465
56	1	0	-2.600637	-2.176094	1.829167
57	1	0	-1.479454	-0.820083	1.590603
58	1	0	-0.929256	-0.854741	-4.579360
59	1	0	3.739577	2.576692	-3.191653
60	1	0	2.405631	2.800401	-2.054025
61	1	0	5.291978	-1.973777	1.314840
62	1	0	3.731749	-2.221235	0.500557
63	1	0	-1.492208	0.595491	-3.703092
64	1	0	0.624893	-4.210165	-0.578567
65	1	0	0.352589	-2.679445	-1.436786
66	1	0	2.462272	0.705883	3.467652
67	1	0	1.166529	1.527128	2.598247
68	6	0	7.616663	2.046333	-0.216195

69	1	0	7.964204	2.470882	-1.167421
70	1	0	7.620954	2.858708	0.527093
71	1	0	8.343654	1.293190	0.114794
72	6	0	-0.286008	-3.395056	4.180839
73	1	0	-0.169547	-4.477918	4.044015
74	1	0	0.172946	-3.111633	5.136867
75	1	0	-1.364394	-3.185974	4.258232
76	6	0	-6.697382	3.074807	1.621525
77	1	0	-6.729866	2.940523	2.710519
78	1	0	-6.817225	4.150028	1.418276
79	1	0	-7.558656	2.557751	1.179424
80	6	0	-2.657816	-4.885908	-2.301285
81	1	0	-1.747045	-5.502003	-2.246804
82	1	0	-3.398443	-5.327223	-1.622396
83	1	0	-3.038954	-4.955703	-3.328472

Final electronic energy: -4158.47483485 a.u.

NIMAG = 0

Final atomic coordinates for **3**$_{\mathbf{C}}$ (two pyramidal P atoms):

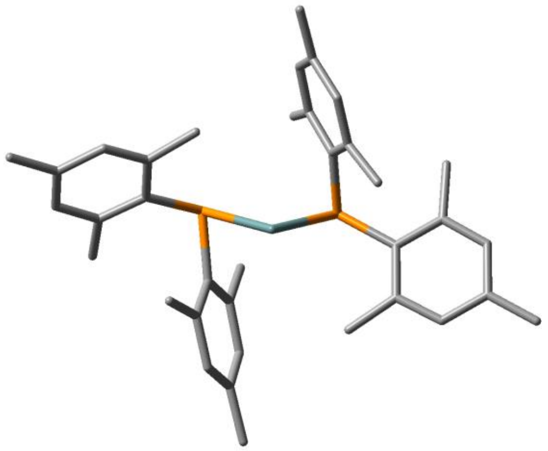

Center Number	Atomic Number	Atomic Type	Coordinates (Angstroms) X	Y	Z
1	15	0	-1.781408	0.221932	1.020361
2	15	0	1.781315	-0.221893	1.020587
3	6	0	-3.380378	-0.172570	0.170401
4	6	0	-4.505453	0.668604	0.378637
5	6	0	-5.736145	0.326478	-0.197595
6	1	0	-6.588569	0.988956	-0.040021
7	6	0	-5.908834	-0.837900	-0.948809
8	6	0	-4.805313	-1.684480	-1.097305
9	1	0	-4.920162	-2.614856	-1.655876
10	6	0	-3.553011	-1.384071	-0.549567
11	6	0	-4.437503	1.927570	1.216931
12	1	0	-3.893748	1.749541	2.155245
13	6	0	-2.434064	-2.386548	-0.749310
14	1	0	-1.724694	-2.056224	-1.522599
15	6	0	-1.356556	1.991701	0.570373
16	6	0	-0.632284	2.725798	1.564253
17	6	0	-0.166156	4.007012	1.274441
18	1	0	0.381866	4.549353	2.045657
19	6	0	-0.377390	4.616940	0.029754
20	6	0	-1.113334	3.915275	-0.921176
21	1	0	-1.308744	4.378580	-1.888673
22	6	0	-1.616411	2.623567	-0.687866
23	6	0	-0.370743	2.165835	2.945131
24	1	0	-1.301513	1.807495	3.405835
25	6	0	-2.473105	2.017807	-1.780251
26	1	0	-2.312062	0.944082	-1.908493
27	6	0	3.380323	0.172626	0.170661

28	6	0	4.505346	-0.668637	0.378849
29	6	0	5.735992	-0.326689	-0.197577
30	1	0	6.588379	-0.989225	-0.040048
31	6	0	5.908691	0.837597	-0.948940
32	6	0	4.805250	1.684290	-1.097334
33	1	0	4.920121	2.614627	-1.655966
34	6	0	3.552994	1.384063	-0.549376
35	6	0	4.437377	-1.927545	1.217234
36	1	0	3.893536	-1.749484	2.155488
37	6	0	2.434154	2.386702	-0.748991
38	1	0	1.724819	2.056646	-1.522429
39	6	0	1.356550	-1.991652	0.570448
40	6	0	0.632210	-2.725787	1.564265
41	6	0	0.166149	-4.007008	1.274403
42	1	0	-0.381928	-4.549375	2.045560
43	6	0	0.377520	-4.616922	0.029723
44	6	0	1.113507	-3.915217	-0.921127
45	1	0	1.309009	-4.378491	-1.888621
46	6	0	1.616520	-2.623481	-0.687760
47	6	0	0.370529	-2.165853	2.945132
48	1	0	1.301262	-1.807595	3.405975
49	6	0	2.473255	-2.017718	-1.780122
50	1	0	2.312354	-0.943965	-1.908265
51	32	0	0.000088	0.000172	-0.609789
52	1	0	-2.843036	-3.352515	-1.071429
53	1	0	-1.852112	-2.552476	0.165175
54	1	0	-5.448168	2.278107	1.459961
55	1	0	-3.907731	2.739384	0.700331
56	1	0	-2.270481	2.518317	-2.734819
57	1	0	-3.539705	2.139757	-1.545467
58	1	0	0.074715	2.934039	3.589517

59	1	0	0.315812	1.309598	2.905392
60	1	0	-0.075062	-2.934052	3.589431
61	1	0	-0.315964	-1.309573	2.905333
62	1	0	2.270524	-2.518115	-2.734727
63	1	0	3.539849	-2.139847	-1.545400
64	1	0	2.843276	3.352693	-1.070851
65	1	0	1.852144	2.552495	0.165473
66	1	0	5.448041	-2.278018	1.460363
67	1	0	3.907686	-2.739420	0.700638
68	6	0	7.252747	1.198751	-1.539980
69	1	0	7.141207	1.636159	-2.541338
70	1	0	7.772592	1.941297	-0.915295
71	1	0	7.901267	0.316723	-1.617337
72	6	0	-7.252941	-1.199271	-1.539599
73	1	0	-7.141510	-1.636754	-2.540937
74	1	0	-7.772590	-1.941828	-0.914764
75	1	0	-7.901582	-0.317330	-1.616927
76	6	0	0.197519	5.981262	-0.265617
77	1	0	-0.238189	6.409657	-1.176609
78	1	0	0.018982	6.675231	0.567141
79	1	0	1.287111	5.919545	-0.408490
80	6	0	-0.197252	-5.981298	-0.265670
81	1	0	0.238117	-6.409409	-1.176957
82	1	0	-0.018158	-6.675415	0.566851
83	1	0	-1.286924	-5.919787	-0.407992

Final electronic energy: -4158.47606962 a.u.

NIMAG = 0

Final atomic coordinates for **3$_D$**:

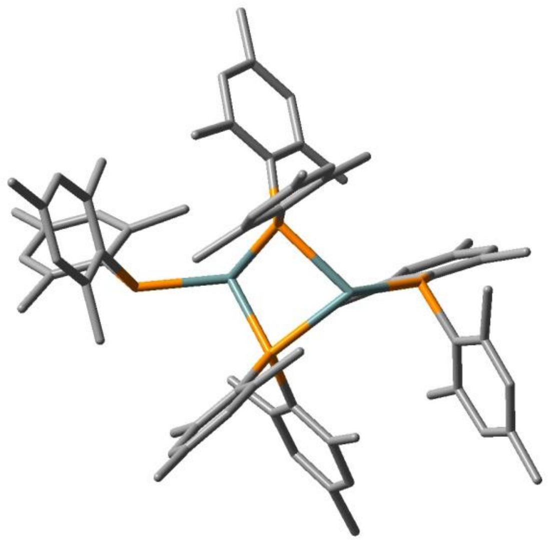

Center Number	Atomic Number	Atomic Type	Coordinates (Angstroms) X	Y	Z
1	15	0	-0.547167	0.090200	1.759731
2	15	0	3.517563	0.857713	1.269475
3	6	0	-1.229610	1.614157	2.571442
4	6	0	-2.545395	1.586104	3.107167
5	6	0	-3.081607	2.765195	3.643230
6	1	0	-4.095071	2.735289	4.044387
7	6	0	-2.376303	3.969077	3.665644
8	6	0	-1.082861	3.975049	3.135904
9	1	0	-0.505138	4.899706	3.142656
10	6	0	-0.496785	2.827810	2.590868
11	6	0	-3.422148	0.355307	3.122279
12	1	0	-3.112422	-0.348182	3.905051
13	1	0	-4.464850	0.637813	3.300889
14	1	0	-3.384255	-0.182157	2.169927
15	6	0	-3.001689	5.232217	4.210634
16	1	0	-3.439385	5.832772	3.398605

17	1	0	-3.804074	5.002179	4.923032
18	1	0	-2.254432	5.858596	4.715267
19	6	0	0.904707	2.925793	2.051489
20	1	0	0.958055	2.543493	1.029565
21	1	0	1.257637	3.962891	2.049294
22	1	0	1.601674	2.327897	2.654287
23	6	0	-0.865040	-1.275220	2.980507
24	6	0	-1.669417	-2.400305	2.701812
25	6	0	-1.776572	-3.417318	3.663106
26	1	0	-2.396701	-4.285090	3.434577
27	6	0	-1.119318	-3.353149	4.889973
28	6	0	-0.326250	-2.227783	5.150510
29	1	0	0.203054	-2.156207	6.101506
30	6	0	-0.177202	-1.191265	4.225830
31	6	0	-2.459369	-2.571322	1.429456
32	1	0	-2.122479	-1.905868	0.632936
33	1	0	-2.387654	-3.603346	1.065913
34	1	0	-3.521137	-2.354233	1.603006
35	6	0	-1.244822	-4.459929	5.911685
36	1	0	-0.260364	-4.885783	6.153274
37	1	0	-1.673223	-4.082078	6.851235
38	1	0	-1.887520	-5.268915	5.543189
39	6	0	0.716051	-0.026330	4.594616
40	1	0	0.153299	0.914858	4.638753
41	1	0	1.182581	-0.199430	5.571631
42	1	0	1.520211	0.117812	3.857568
43	6	0	3.954113	2.541094	0.616635
44	6	0	3.254209	3.256878	-0.378042
45	6	0	3.638345	4.569919	-0.689446
46	1	0	3.084672	5.105999	-1.462240
47	6	0	4.713425	5.198406	-0.063279

48	6	0	5.421075	4.468697	0.900143
49	1	0	6.276104	4.929674	1.396910
50	6	0	5.063580	3.166199	1.259244
51	6	0	2.137818	2.662603	-1.195851
52	1	0	1.244109	3.300252	-1.164824
53	1	0	2.446410	2.573381	-2.246231
54	1	0	1.852470	1.666254	-0.849978
55	6	0	5.098260	6.621894	-0.396489
56	1	0	4.743409	7.317816	0.379082
57	1	0	6.189331	6.733587	-0.458584
58	1	0	4.661490	6.936878	-1.352690
59	6	0	5.888886	2.464602	2.317893
60	1	0	6.375840	1.565009	1.917276
61	1	0	6.663909	3.136054	2.708042
62	1	0	5.255628	2.130959	3.152236
63	6	0	4.863143	-0.286134	0.683203
64	6	0	5.324582	-1.279163	1.594002
65	6	0	6.285841	-2.207463	1.174002
66	1	0	6.625135	-2.964568	1.882234
67	6	0	6.832851	-2.182549	-0.111214
68	6	0	6.404377	-1.175615	-0.979752
69	1	0	6.828993	-1.122846	-1.983112
70	6	0	5.433991	-0.233027	-0.617755
71	6	0	4.837202	-1.368451	3.027400
72	1	0	3.797829	-1.718223	3.081280
73	1	0	5.464568	-2.064773	3.598735
74	1	0	4.859112	-0.383029	3.511694
75	6	0	7.838108	-3.221169	-0.553592
76	1	0	8.444766	-3.572221	0.291488
77	1	0	7.332461	-4.100712	-0.982137
78	1	0	8.511281	-2.820847	-1.322859

79	6	0	5.041725	0.797356	-1.649301
80	1	0	5.388508	1.798905	-1.367974
81	1	0	5.471815	0.538955	-2.622415
82	1	0	3.954685	0.847818	-1.764775
83	1	0	-5.738323	6.301138	-3.148978
84	6	0	-5.923816	5.408427	-3.760035
85	6	0	-5.220331	4.199171	-3.187500
86	1	0	-5.570491	5.623647	-4.780217
87	1	0	-7.008510	5.245896	-3.818325
88	6	0	-4.138999	4.331464	-2.317345
89	6	0	-5.617499	2.903955	-3.526621
90	6	0	-3.456307	3.229297	-1.779037
91	1	0	-3.819661	5.332345	-2.023774
92	1	0	-6.462668	2.765629	-4.202421
93	6	0	-4.962607	1.768920	-3.036718
94	6	0	-3.860771	1.914702	-2.138125
95	6	0	-2.341367	3.550397	-0.808769
96	6	0	-5.480693	0.420252	-3.496185
97	15	0	-3.099001	0.294139	-1.625183
98	1	0	-1.350829	3.360901	-1.244787
99	1	0	-2.381356	4.608470	-0.520820
100	1	0	-2.399059	2.956433	0.111501
101	1	0	-5.937504	-0.142619	-2.671804
102	1	0	-6.232092	0.550609	-4.284833
103	1	0	-4.664212	-0.204235	-3.886020
104	6	0	-4.416879	-0.511567	-0.597676
105	15	0	0.534777	-1.229198	-1.125907
106	6	0	-4.653866	-1.898578	-0.816097
107	6	0	-5.249094	0.184510	0.318792
108	6	0	1.708974	-0.855165	-2.513937
109	6	0	-0.057830	-2.967983	-1.499261

110	6	0	-5.685478	-2.544503	-0.129330
111	6	0	-3.800937	-2.721074	-1.751134
112	6	0	-6.271314	-0.511265	0.981692
113	6	0	-5.088139	1.650165	0.642589
114	6	0	2.875905	-1.669222	-2.603838
115	6	0	1.508367	0.166614	-3.481240
116	6	0	0.076502	-4.038941	-0.574478
117	6	0	-0.671881	-3.227931	-2.758246
118	1	0	-5.845617	-3.608300	-0.310312
119	6	0	-6.509041	-1.870988	0.778637
120	1	0	-2.778276	-2.817689	-1.370361
121	1	0	-4.209769	-3.732776	-1.863680
122	1	0	-3.723348	-2.255048	-2.741388
123	1	0	-6.897031	0.034975	1.688857
124	1	0	-5.485175	2.287415	-0.157019
125	1	0	-5.616126	1.893156	1.572445
126	1	0	-4.035352	1.916488	0.772353
127	6	0	3.788122	-1.449612	-3.639820
128	6	0	3.203703	-2.773177	-1.623517
129	6	0	2.478928	0.361828	-4.475870
130	6	0	0.289567	1.060536	-3.547491
131	6	0	-0.421131	-5.306013	-0.912794
132	6	0	0.771990	-3.919399	0.759312
133	6	0	-1.115265	-4.522103	-3.054770
134	6	0	-0.938620	-2.161075	-3.791645
135	6	0	-7.605246	-2.600395	1.521193
136	1	0	4.675331	-2.080228	-3.689448
137	6	0	3.621683	-0.430288	-4.577910
138	1	0	2.453939	-3.572649	-1.649393
139	1	0	4.184510	-3.202793	-1.852758
140	1	0	3.254185	-2.392196	-0.595626

141	1	0	2.316449	1.153219	-5.208161
142	1	0	0.315152	1.838851	-2.775955
143	1	0	0.243534	1.561906	-4.522191
144	1	0	-0.642530	0.503075	-3.417061
145	1	0	-0.315946	-6.113005	-0.187337
146	6	0	-1.016908	-5.574965	-2.144877
147	1	0	0.312851	-3.170644	1.410517
148	1	0	0.741385	-4.877249	1.292381
149	1	0	1.822285	-3.632499	0.630733
150	1	0	-1.580298	-4.698605	-4.024950
151	1	0	-0.012344	-1.744918	-4.201775
152	1	0	-1.526777	-2.574945	-4.619304
153	1	0	-1.516696	-1.339934	-3.348042
154	1	0	-8.240396	-3.170402	0.828493
155	1	0	-7.184868	-3.318900	2.240761
156	1	0	-8.242238	-1.900344	2.076134
157	6	0	4.667854	-0.167503	-5.634411
158	6	0	-1.563434	-6.944655	-2.472740
159	1	0	5.465333	0.478297	-5.234708
160	1	0	5.138496	-1.101107	-5.969074
161	1	0	4.236853	0.340558	-6.506360
162	1	0	-2.646819	-6.987884	-2.282673
163	1	0	-1.407370	-7.190748	-3.531277
164	1	0	-1.087007	-7.719881	-1.859402
165	32	0	1.769534	-0.778117	1.149253
166	32	0	-0.955371	0.639724	-0.556499

Final electronic energy: -8316.99243565 a.u.

NIMAG = 0

Final atomic coordinates for $\mathbf{3_T}$:

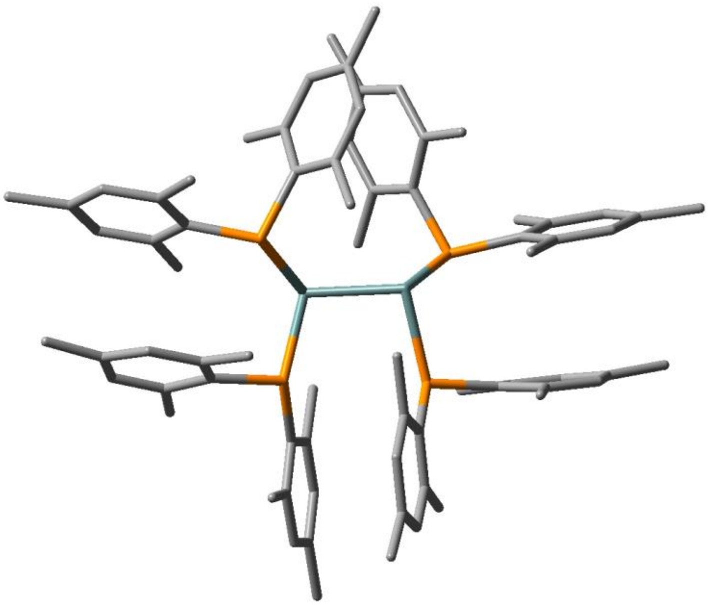

Center Number	Atomic Number	Atomic Type	Coordinates (Angstroms) X	Y	Z
1	32	0	-0.406109	0.306976	0.839450
2	32	0	1.604837	1.279098	2.036307
3	15	0	-1.823962	-0.353622	2.611707
4	15	0	0.496037	-1.852290	0.373738
5	15	0	3.299034	0.298422	0.673232
6	15	0	1.764706	3.465348	1.152819
7	6	0	-2.053366	1.171222	3.639070
8	6	0	-2.971653	2.208647	3.337972
9	6	0	-3.152941	3.249442	4.254856
10	6	0	-2.430318	3.328286	5.446814
11	6	0	-1.494580	2.324755	5.710782
12	6	0	-1.298355	1.245827	4.841489
13	6	0	-3.713043	2.278569	2.026460
14	6	0	-2.647245	4.470252	6.411495
15	6	0	-0.272431	0.198979	5.216892

16	6	0	-3.521110	-0.924332	2.135068
17	6	0	-4.071714	-0.949178	0.828039
18	6	0	-5.331135	-1.528237	0.624615
19	6	0	-6.080507	-2.080110	1.664983
20	6	0	-5.535663	-2.034996	2.950039
21	6	0	-4.276514	-1.479596	3.205709
22	6	0	-3.382019	-0.357309	-0.375326
23	6	0	-7.419055	-2.729921	1.400904
24	6	0	-3.766602	-1.504771	4.631829
25	6	0	-0.902534	-3.054123	0.442796
26	6	0	-1.768265	-3.337165	-0.644942
27	6	0	-2.911440	-4.114228	-0.419695
28	6	0	-3.220029	-4.641944	0.835857
29	6	0	-2.302991	-4.440557	1.871004
30	6	0	-1.145627	-3.675504	1.699831
31	6	0	-1.511399	-2.843388	-2.051372
32	6	0	-4.507686	-5.389718	1.082721
33	6	0	-0.185769	-3.554887	2.864895
34	6	0	1.369454	-2.078980	-1.234875
35	6	0	1.489384	-1.111418	-2.266363
36	6	0	2.259323	-1.406581	-3.399442
37	6	0	2.922437	-2.625450	-3.557502
38	6	0	2.799123	-3.567258	-2.534050
39	6	0	2.047648	-3.321798	-1.379696
40	6	0	0.816482	0.236176	-2.219410
41	6	0	3.739744	-2.914849	-4.795121
42	6	0	2.011728	-4.393743	-0.313230
43	6	0	3.959911	-1.343575	1.190606
44	6	0	4.821157	-1.969255	0.245640
45	6	0	5.322466	-3.248196	0.510267
46	6	0	4.995965	-3.950980	1.672073

47	6	0	4.149697	-3.326656	2.590708
48	6	0	3.622880	-2.045596	2.377104
49	6	0	5.205052	-1.311052	-1.060891
50	6	0	5.499344	-5.356069	1.904655
51	6	0	2.726807	-1.479323	3.448186
52	6	0	4.704708	1.490999	0.598332
53	6	0	4.693741	2.420211	-0.479475
54	6	0	5.614641	3.471820	-0.483581
55	6	0	6.570146	3.626331	0.524492
56	6	0	6.635563	2.645253	1.516100
57	6	0	5.735457	1.573617	1.569559
58	6	0	3.728883	2.306185	-1.641080
59	6	0	7.483717	4.827712	0.541228
60	6	0	5.917466	0.547022	2.665298
61	6	0	3.014229	4.580072	1.944155
62	6	0	3.210399	5.816907	1.268271
63	6	0	4.196453	6.703047	1.717432
64	6	0	5.016823	6.410038	2.808918
65	6	0	4.811402	5.195233	3.465177
66	6	0	3.831090	4.278359	3.063878
67	6	0	2.391380	6.220759	0.059739
68	6	0	6.109712	7.356908	3.247797
69	6	0	3.691737	3.017107	3.878744
70	6	0	0.108777	4.241531	1.448558
71	6	0	-0.766580	4.312035	0.330017
72	6	0	-1.990696	4.978372	0.455718
73	6	0	-2.377085	5.599955	1.645954
74	6	0	-1.509523	5.517460	2.736655
75	6	0	-0.290699	4.833945	2.673223
76	6	0	-0.424348	3.685278	-1.003849
77	6	0	-3.717715	6.283038	1.773497

78	6	0	0.517585	4.703022	3.939736
79	1	0	-8.032855	-2.759590	2.310209
80	1	0	-7.288475	-3.766243	1.052980
81	1	0	-7.975279	-2.191498	0.622099
82	1	0	-3.126973	0.697659	-0.213724
83	1	0	-4.025332	-0.423467	-1.261310
84	1	0	-2.456112	-0.892506	-0.600251
85	1	0	-2.796807	-2.019753	4.690069
86	1	0	-4.478465	-2.025877	5.283759
87	1	0	-3.611727	-0.491487	5.025681
88	1	0	-6.101829	-2.451343	3.784175
89	1	0	-5.740203	-1.539761	-0.386261
90	1	0	-1.218991	-1.788220	-2.069277
91	1	0	-2.408837	-2.969342	-2.669479
92	1	0	-0.685244	-3.396515	-2.517888
93	1	0	-4.330464	-6.315666	1.647327
94	1	0	-5.007419	-5.646618	0.140100
95	1	0	-5.193816	-4.766853	1.674468
96	1	0	0.845330	-3.761519	2.550623
97	1	0	-0.464033	-4.260781	3.658213
98	1	0	-0.204683	-2.542331	3.288885
99	1	0	-2.493602	-4.888106	2.847068
100	1	0	-3.592369	-4.293989	-1.252227
101	1	0	0.954777	0.772880	-3.165898
102	1	0	-0.262884	0.143563	-2.032058
103	1	0	1.238753	0.853660	-1.418264
104	1	0	2.345623	-0.649760	-4.179840
105	1	0	4.233430	-2.006492	-5.164866
106	1	0	3.103773	-3.297761	-5.608051
107	1	0	4.509106	-3.671124	-4.592689
108	1	0	3.321921	-4.520261	-2.621306

109	1	0	2.349989	-3.990962	0.650591
110	1	0	2.668870	-5.227286	-0.588836
111	1	0	0.995739	-4.784589	-0.166380
112	1	0	5.802840	-1.997180	-1.672703
113	1	0	4.308031	-1.034717	-1.630766
114	1	0	5.785472	-0.392974	-0.897376
115	1	0	5.975991	-3.713319	-0.228476
116	1	0	6.459838	-5.523212	1.400295
117	1	0	5.626691	-5.560902	2.975708
118	1	0	4.785069	-6.095795	1.510961
119	1	0	3.882794	-3.851844	3.508417
120	1	0	3.066294	-0.487642	3.779732
121	1	0	1.700899	-1.369746	3.077729
122	1	0	2.699380	-2.141464	4.322202
123	1	0	6.612084	0.921659	3.427172
124	1	0	4.969716	0.292187	3.151159
125	1	0	6.315686	-0.393511	2.261556
126	1	0	7.400332	2.723324	2.289475
127	1	0	6.915855	5.724785	0.826855
128	1	0	7.918854	5.012977	-0.450715
129	1	0	8.301379	4.697797	1.261487
130	1	0	5.578425	4.194596	-1.299437
131	1	0	4.018168	2.999449	-2.441576
132	1	0	3.713710	1.285123	-2.043314
133	1	0	2.705192	2.553472	-1.330786
134	1	0	2.745593	7.178892	-0.340019
135	1	0	2.465240	5.461895	-0.732488
136	1	0	1.325774	6.319293	0.305911
137	1	0	4.328522	7.648290	1.189495
138	1	0	6.215356	7.362384	4.340793
139	1	0	5.906304	8.381815	2.912154

140	1	0	7.080901	7.054795	2.826430
141	1	0	5.431077	4.948558	4.328026
142	1	0	4.375514	3.033211	4.736273
143	1	0	2.669061	2.892369	4.257038
144	1	0	3.934504	2.134937	3.280950
145	1	0	0.493569	4.115649	-1.425700
146	1	0	-0.253405	2.603095	-0.900865
147	1	0	-1.242971	3.833516	-1.719446
148	1	0	-2.656242	5.018320	-0.407465
149	1	0	-4.092086	6.615710	0.797038
150	1	0	-4.465976	5.594016	2.196986
151	1	0	-3.660703	7.152693	2.441278
152	1	0	-1.800741	5.970466	3.684019
153	1	0	-0.042149	5.099761	4.794059
154	1	0	0.721855	3.643492	4.136079
155	1	0	1.480943	5.222136	3.875924
156	1	0	-2.996817	2.237404	1.196790
157	1	0	-4.263176	3.222725	1.945881
158	1	0	-4.416665	1.447859	1.900551
159	1	0	-3.855194	4.044214	4.005182
160	1	0	-1.750598	4.654033	7.017362
161	1	0	-3.476270	4.253851	7.102585
162	1	0	-2.901347	5.395105	5.876581
163	1	0	-0.898152	2.378139	6.622366
164	1	0	0.210765	0.457953	6.167483
165	1	0	-0.730316	-0.794218	5.314569
166	1	0	0.510090	0.117579	4.447686

Final electronic energy: -8317.02132570

NIMAG = 0

Final atomic coordinates for **7**:

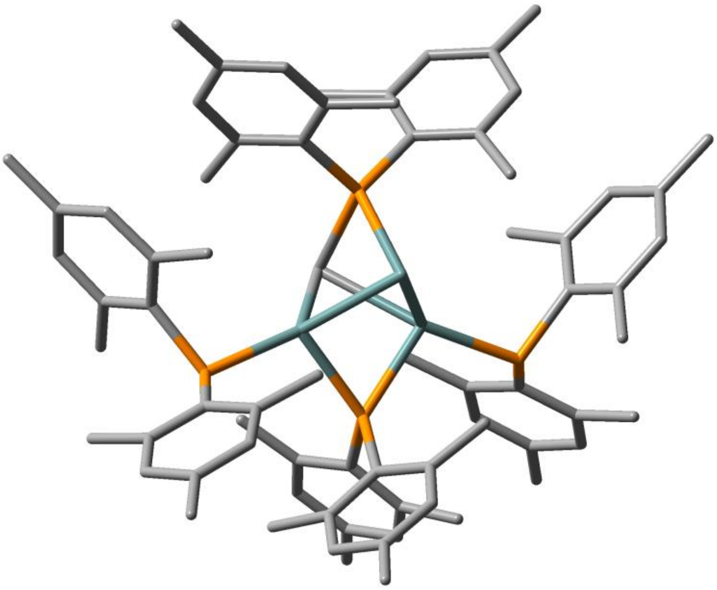

Center Number	Atomic Number	Atomic Type	Coordinates (Angstroms) X	Y	Z
1	32	0	-1.477066	0.222679	0.201484
2	32	0	1.469369	0.218808	-0.204462
3	32	0	-0.244486	-0.961650	-1.809572
4	32	0	0.232563	-0.964296	1.804604
5	15	0	-3.617719	1.050538	0.745339
6	15	0	3.612432	1.044351	-0.743605
7	15	0	-0.003510	-2.695053	-0.004439
8	15	0	-0.001475	2.122775	-0.002644
9	6	0	-4.528174	-0.427871	1.403131
10	6	0	-5.043548	-1.466464	0.586693
11	6	0	-5.836839	-2.461160	1.167273
12	1	0	-6.225611	-3.253518	0.527953
13	6	0	-6.106562	-2.493425	2.538305
14	6	0	-5.536999	-1.501517	3.340640
15	1	0	-5.702556	-1.527092	4.418193

16	6	0	-4.754893	-0.471251	2.803135
17	6	0	-4.707049	-1.581690	-0.878231
18	1	0	-5.139968	-0.761684	-1.463784
19	1	0	-5.063713	-2.536288	-1.281213
20	1	0	-3.618234	-1.543274	-1.016978
21	6	0	-6.969522	-3.584825	3.128409
22	1	0	-8.027418	-3.437477	2.864332
23	1	0	-6.894684	-3.603887	4.222817
24	1	0	-6.675636	-4.572156	2.744356
25	6	0	-4.151040	0.542501	3.752463
26	1	0	-3.054349	0.525379	3.704323
27	1	0	-4.453523	0.327447	4.784972
28	1	0	-4.459683	1.565244	3.498888
29	6	0	-4.669024	1.683455	-0.660748
30	6	0	-5.862074	2.334226	-0.225741
31	6	0	-6.671481	2.996435	-1.155713
32	1	0	-7.580194	3.484391	-0.800632
33	6	0	-6.347275	3.059611	-2.512985
34	6	0	-5.186758	2.407545	-2.928405
35	1	0	-4.919914	2.421444	-3.985613
36	6	0	-4.343870	1.720235	-2.042118
37	6	0	-6.303150	2.354666	1.224435
38	1	0	-6.502058	1.344432	1.605003
39	1	0	-7.215333	2.953252	1.337148
40	1	0	-5.521857	2.785558	1.867548
41	6	0	-7.207707	3.826162	-3.491149
42	1	0	-8.264536	3.803909	-3.194649
43	1	0	-7.121087	3.411466	-4.503936
44	1	0	-6.901626	4.882783	-3.539345
45	6	0	-3.135493	1.044908	-2.637165
46	1	0	-2.207002	1.498769	-2.280835

47	1	0	-3.146610	1.134373	-3.729906
48	1	0	-3.093298	-0.017193	-2.379539
49	6	0	4.531655	-0.432255	-1.393380
50	6	0	4.766981	-0.477348	-2.794324
51	6	0	5.558090	-1.501138	-3.325020
52	1	0	5.727367	-1.527124	-4.402226
53	6	0	6.132919	-2.487687	-2.515669
54	6	0	5.857157	-2.453036	-1.148321
55	1	0	6.251010	-3.240861	-0.506790
56	6	0	5.049656	-1.463450	-0.573215
57	6	0	4.159063	0.530498	-3.747374
58	1	0	3.062397	0.508729	-3.699886
59	1	0	4.463267	0.313412	-4.778956
60	1	0	4.462971	1.555442	-3.497030
61	6	0	7.002807	-3.571043	-3.110813
62	1	0	6.521469	-4.030368	-3.985814
63	1	0	7.210470	-4.359476	-2.376407
64	1	0	7.966907	-3.162867	-3.448969
65	6	0	4.705654	-1.581258	0.889632
66	1	0	5.126002	-0.756535	1.477651
67	1	0	5.070479	-2.531485	1.295662
68	1	0	3.615567	-1.555715	1.021224
69	6	0	4.655756	1.684746	0.664978
70	6	0	5.846183	2.342142	0.232546
71	6	0	6.648039	3.011655	1.163705
72	1	0	7.554739	3.504772	0.810594
73	6	0	6.319048	3.075402	2.519882
74	6	0	5.161975	2.416057	2.933004
75	1	0	4.891893	2.429932	3.989375
76	6	0	4.326433	1.721492	2.045300
77	6	0	6.292438	2.361606	-1.216100

78	1	0	6.497393	1.351476	-1.593761
79	1	0	7.202383	2.963999	-1.326683
80	1	0	5.511666	2.787606	-1.863075
81	6	0	7.171462	3.849700	3.498960
82	1	0	8.230430	3.826885	3.210149
83	1	0	7.078198	3.441705	4.513859
84	1	0	6.863812	4.906241	3.537716
85	6	0	3.120923	1.039015	2.637989
86	1	0	2.190725	1.491450	2.284286
87	1	0	3.132454	1.123408	3.731146
88	1	0	3.081934	-0.021799	2.375127
89	6	0	1.489000	-3.808388	0.017223
90	6	0	2.442440	-3.843896	-1.034784
91	6	0	3.555261	-4.687641	-0.919173
92	1	0	4.275303	-4.701125	-1.734980
93	6	0	3.783851	-5.482536	0.202802
94	6	0	2.840866	-5.437119	1.231606
95	1	0	2.988675	-6.052396	2.119887
96	6	0	1.703319	-4.624464	1.163245
97	6	0	2.354246	-3.009143	-2.290243
98	1	0	1.394971	-3.134790	-2.801797
99	1	0	3.158696	-3.278388	-2.983172
100	1	0	2.471151	-1.944273	-2.064690
101	6	0	5.028733	-6.331436	0.313269
102	1	0	4.855066	-7.215768	0.939829
103	1	0	5.851087	-5.759595	0.771103
104	1	0	5.370673	-6.663831	-0.675475
105	6	0	0.752415	-4.650344	2.336853
106	1	0	0.663035	-3.651693	2.787865
107	1	0	1.113506	-5.340054	3.108891
108	1	0	-0.253278	-4.963896	2.033051

109	6	0	-1.482608	-3.826441	-0.025790
110	6	0	-2.438008	-3.868113	1.024103
111	6	0	-3.540534	-4.725825	0.910258
112	1	0	-4.266978	-4.736934	1.720568
113	6	0	-3.753064	-5.534627	-0.205079
114	6	0	-2.813154	-5.475541	-1.236054
115	1	0	-2.956832	-6.089106	-2.126161
116	6	0	-1.687436	-4.646500	-1.170621
117	6	0	-2.362807	-3.023713	2.273860
118	1	0	-1.407954	-3.146791	2.794712
119	1	0	-3.173208	-3.288672	2.961272
120	1	0	-2.477202	-1.960205	2.041115
121	6	0	-4.950678	-6.452733	-0.285039
122	1	0	-4.714389	-7.444977	0.128810
123	1	0	-5.271498	-6.597586	-1.324836
124	1	0	-5.795952	-6.050093	0.288319
125	6	0	-0.741665	-4.655433	-2.348407
126	1	0	-0.668089	-3.654336	-2.796811
127	1	0	-1.096010	-5.348255	-3.120773
128	1	0	0.269367	-4.955988	-2.049228
129	6	0	0.642254	3.236389	1.333664
130	6	0	1.693690	4.135593	0.993057
131	6	0	2.350403	4.829815	2.012532
132	1	0	3.173950	5.490345	1.741447
133	6	0	2.002214	4.688437	3.358198
134	6	0	0.928183	3.854685	3.665526
135	1	0	0.615132	3.751339	4.704947
136	6	0	0.238259	3.124761	2.686338
137	6	0	2.146522	4.391302	-0.426899
138	1	0	2.376474	3.460771	-0.957509
139	1	0	3.055216	5.004254	-0.426687

140	1	0	1.371597	4.910290	-1.005443
141	6	0	2.786883	5.397544	4.436005
142	1	0	3.758280	4.903178	4.587925
143	1	0	2.249150	5.390235	5.392350
144	1	0	2.992370	6.440209	4.158228
145	6	0	-0.883140	2.233391	3.163507
146	1	0	-1.713498	2.174183	2.455490
147	1	0	-1.273737	2.603313	4.120025
148	1	0	-0.527912	1.205791	3.333265
149	6	0	-0.644441	3.233685	-1.341623
150	6	0	-0.240581	3.119086	-2.694029
151	6	0	-0.932170	3.845028	-3.675066
152	1	0	-0.619180	3.739355	-4.714278
153	6	0	-2.007767	4.677451	-3.369823
154	6	0	-2.355266	4.822640	-2.024329
155	1	0	-3.179457	5.482979	-1.754747
156	6	0	-1.696625	4.132911	-1.003102
157	6	0	0.882675	2.229025	-3.169401
158	1	0	1.710541	2.167899	-2.458710
159	1	0	1.276552	2.601522	-4.123605
160	1	0	0.527541	1.202115	-3.343130
161	6	0	-2.794336	5.381846	-4.449344
162	1	0	-2.258396	5.370074	-5.406644
163	1	0	-2.999028	6.425825	-4.175911
164	1	0	-3.766185	4.887118	-4.597157
165	6	0	-2.147811	4.393664	0.416509
166	1	0	-2.376812	3.465235	0.951204
167	1	0	-3.056653	5.006402	0.415165
168	1	0	-1.372242	4.915170	0.991925

Final electronic energy: -12473.09306830

NIMAG = 0

Final atomic coordinates for **6**:

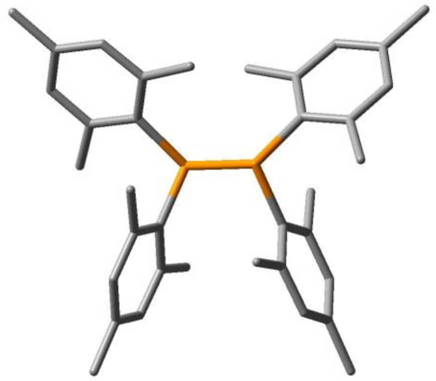

Center Number	Atomic Number	Atomic Type	Coordinates (Angstroms) X	Y	Z
1	15	0	-0.746840	0.855593	-0.314791
2	6	0	-0.282453	2.163497	-1.571177
3	6	0	0.656446	2.079158	-2.636354
4	6	0	0.791721	3.161912	-3.517651
5	6	0	0.047993	4.336511	-3.395563
6	6	0	-0.873040	4.405916	-2.350704
7	6	0	-1.064098	3.350555	-1.450350
8	6	0	1.578174	0.902202	-2.883407
9	6	0	0.210680	5.473043	-4.378612
10	6	0	-2.112779	3.550592	-0.372183
11	6	0	-0.193276	1.623576	1.301040
12	6	0	1.002071	2.369859	1.466828
13	6	0	1.272103	2.966178	2.706986
14	6	0	0.409718	2.844412	3.797446
15	6	0	-0.765849	2.110731	3.618904
16	6	0	-1.088609	1.504672	2.399165

17	6	0	2.034808	2.546540	0.376850
18	6	0	0.746840	3.460999	5.135509
19	6	0	-2.398895	0.748749	2.320670
20	1	0	1.521718	3.083205	-4.324149
21	1	0	-1.475807	5.307033	-2.230292
22	1	0	2.196922	3.532820	2.822246
23	1	0	-1.461242	2.008135	4.452706
24	1	0	2.153842	1.065657	-3.802734
25	1	0	2.292740	0.766203	-2.063082
26	1	0	1.044905	-0.046875	-3.001798
27	1	0	-0.045884	6.435665	-3.917748
28	1	0	1.241428	5.529036	-4.752455
29	1	0	-0.448503	5.336817	-5.249714
30	1	0	-2.709143	4.445647	-0.587245
31	1	0	-2.792376	2.688912	-0.308229
32	1	0	-1.658153	3.668601	0.619862
33	1	0	2.988643	2.870666	0.811342
34	1	0	2.200339	1.608880	-0.161398
35	1	0	1.718876	3.295823	-0.359691
36	1	0	1.456023	4.290879	5.023298
37	1	0	-0.154662	3.836643	5.637323
38	1	0	1.207272	2.716806	5.803232
39	1	0	-2.995924	0.933026	3.222528
40	1	0	-2.984804	1.045434	1.441828
41	1	0	-2.224872	-0.330208	2.241657
42	15	0	0.746840	-0.855593	-0.314791
43	6	0	0.282453	-2.163497	-1.571177
44	6	0	0.193276	-1.623576	1.301040
45	6	0	-0.656446	-2.079158	-2.636354
46	6	0	1.064098	-3.350555	-1.450350
47	6	0	-1.002071	-2.369859	1.466828

48	6	0	1.088609	-1.504672	2.399165
49	6	0	-0.791721	-3.161912	-3.517651
50	6	0	-1.578174	-0.902202	-2.883407
51	6	0	0.873040	-4.405916	-2.350704
52	6	0	2.112779	-3.550592	-0.372183
53	6	0	-1.272103	-2.966178	2.706986
54	6	0	-2.034808	-2.546540	0.376850
55	6	0	0.765849	-2.110731	3.618904
56	6	0	2.398895	-0.748749	2.320670
57	6	0	-0.047993	-4.336511	-3.395563
58	1	0	-1.521718	-3.083205	-4.324149
59	1	0	-2.153842	-1.065657	-3.802734
60	1	0	-2.292740	-0.766203	-2.063082
61	1	0	-1.044905	0.046875	-3.001798
62	1	0	1.475807	-5.307033	-2.230292
63	1	0	2.709143	-4.445647	-0.587245
64	1	0	2.792376	-2.688912	-0.308229
65	1	0	1.658153	-3.668601	0.619862
66	6	0	-0.409718	-2.844412	3.797446
67	1	0	-2.196922	-3.532820	2.822246
68	1	0	-2.988643	-2.870666	0.811342
69	1	0	-2.200339	-1.608880	-0.161398
70	1	0	-1.718876	-3.295823	-0.359691
71	1	0	1.461242	-2.008135	4.452706
72	1	0	2.995924	-0.933026	3.222528
73	1	0	2.984804	-1.045434	1.441828
74	1	0	2.224872	0.330208	2.241657
75	6	0	-0.210680	-5.473043	-4.378612
76	6	0	-0.746840	-3.460999	5.135509
77	1	0	0.045884	-6.435665	-3.917748
78	1	0	-1.241428	-5.529036	-4.752455

79	1	0	0.448503	-5.336817	-5.249714
80	1	0	-1.456023	-4.290879	5.023298
81	1	0	0.154662	-3.836643	5.637323
82	1	0	-1.207272	-2.716806	5.803232

Final electronic energy: -2080.45439698 a.u.

NIMAG = 0

Final atomic coordinates for **9**:

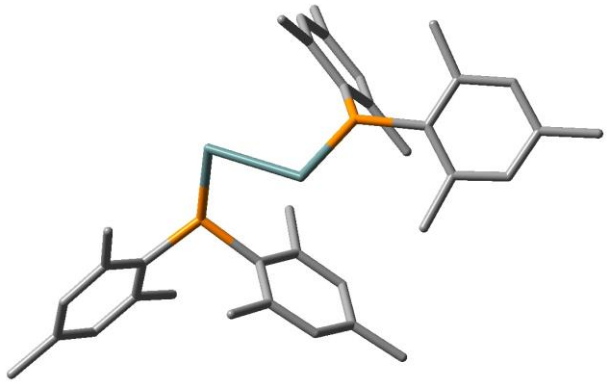

Center Number	Atomic Number	Atomic Type	Coordinates (Angstroms) X	Y	Z
1	15	0	2.228951	0.382125	1.019399
2	6	0	3.705192	-0.688845	0.795575
3	6	0	3.625339	-2.072955	0.488570
4	6	0	4.811563	-2.793014	0.292587
5	1	0	4.741119	-3.852300	0.042897
6	6	0	6.073725	-2.203538	0.393408
7	6	0	6.134377	-0.842906	0.708301
8	1	0	7.107457	-0.358961	0.800550
9	6	0	4.983550	-0.075852	0.918356

10	6	0	2.322293	-2.822221	0.337799
11	1	0	1.840610	-2.988076	1.310669
12	1	0	2.497488	-3.800994	-0.125849
13	1	0	1.599747	-2.270212	-0.275727
14	6	0	7.333986	-3.015185	0.200685
15	1	0	7.729564	-3.360346	1.168177
16	1	0	8.120352	-2.418871	-0.280506
17	1	0	7.143242	-3.903434	-0.415063
18	6	0	5.157726	1.386197	1.270940
19	1	0	4.852957	2.040673	0.443966
20	1	0	6.205936	1.598392	1.513497
21	1	0	4.537514	1.659426	2.136043
22	6	0	2.359571	1.725132	-0.229524
23	6	0	2.183822	3.073836	0.175338
24	6	0	2.275209	4.090340	-0.783063
25	1	0	2.138389	5.123641	-0.462570
26	6	0	2.552422	3.823684	-2.126529
27	6	0	2.747442	2.491149	-2.502045
28	1	0	2.968068	2.259076	-3.544668
29	6	0	2.654651	1.434910	-1.588529
30	6	0	1.914904	3.466491	1.609729
31	1	0	0.901648	3.174674	1.922741
32	1	0	2.005454	4.552535	1.733219
33	1	0	2.610204	2.970136	2.298906
34	6	0	2.613032	4.937280	-3.146347
35	1	0	3.327050	4.704967	-3.947160
36	1	0	2.907008	5.886374	-2.680018
37	1	0	1.629180	5.090304	-3.615750
38	6	0	2.851949	0.027595	-2.103082
39	1	0	3.825187	-0.379537	-1.801158
40	1	0	2.795099	0.013815	-3.198376

41	1	0	2.083928	-0.646264	-1.706150
42	15	0	-2.449668	0.559461	0.009429
43	6	0	-4.282823	0.379555	-0.040382
44	6	0	-5.096948	0.418796	1.123361
45	6	0	-6.475694	0.195169	0.996579
46	1	0	-7.089471	0.213577	1.897515
47	6	0	-7.085940	-0.054808	-0.233771
48	6	0	-6.274152	-0.068966	-1.371985
49	1	0	-6.726373	-0.249557	-2.347944
50	6	0	-4.895106	0.152007	-1.305525
51	6	0	-4.560676	0.673212	2.512782
52	1	0	-4.207302	1.705782	2.627692
53	1	0	-5.339732	0.490817	3.263420
54	1	0	-3.702229	0.022254	2.731813
55	6	0	-8.577844	-0.270959	-0.340304
56	1	0	-9.083295	0.644530	-0.683523
57	1	0	-8.812221	-1.063440	-1.063573
58	1	0	-9.009143	-0.546038	0.630387
59	6	0	-4.111415	0.150817	-2.598879
60	1	0	-3.413703	-0.694659	-2.651952
61	1	0	-4.789602	0.095886	-3.458735
62	1	0	-3.511874	1.067135	-2.686347
63	6	0	-1.741093	-0.994669	-0.685908
64	6	0	-0.838943	-0.906114	-1.781158
65	6	0	-0.265211	-2.077060	-2.284375
66	1	0	0.422785	-2.000373	-3.126645
67	6	0	-0.542848	-3.337140	-1.739504
68	6	0	-1.425159	-3.405697	-0.660013
69	1	0	-1.648300	-4.375889	-0.215343
70	6	0	-2.033707	-2.262841	-0.121107
71	6	0	-0.469170	0.415079	-2.415266

72	1	0	0.051047	1.063567	-1.701544
73	1	0	0.191035	0.256661	-3.275308
74	1	0	-1.359547	0.960777	-2.750142
75	6	0	0.123140	-4.574922	-2.292174
76	1	0	-0.290454	-5.484914	-1.840593
77	1	0	-0.003440	-4.639599	-3.381903
78	1	0	1.204411	-4.555877	-2.090862
79	6	0	-2.967674	-2.419277	1.054977
80	1	0	-3.994994	-2.136235	0.791865
81	1	0	-2.966875	-3.454744	1.415053
82	1	0	-2.659139	-1.761752	1.879585
83	32	0	-1.329516	1.468227	1.752545
84	32	0	0.323423	-0.483927	1.813389

Final electronic energy: -6236.48263952 a.u.

NIMAG = 0

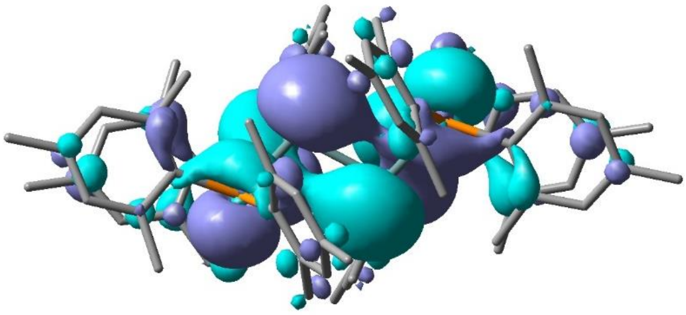

Figure S9. Alternative view of the HOMO of **3**.

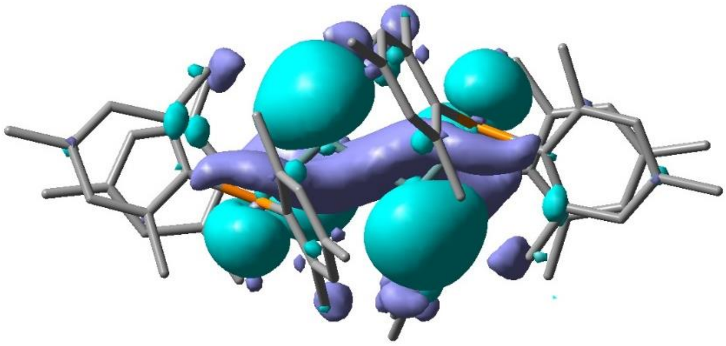

Figure S10. Alternative view of the LUMO of **3**.

Table S1. Comparison of key bond lengths (Å) and angles (°) (experimental and calculated) for **3**.

	X-ray	Calcd. (B97D/6-311G(2d,p)
Ge(1)-Ge(2)	2.4476(9)	2.5341
Ge(1)-P(1)	2.3318(16)	2.3854
Ge(1)-P(2)	2.3176(16)	2.3643
Ge(2)-P(3)	2.3483(15)	2.3639
Ge(2)-P(4)	2.3753(16)	2.3860
P(1)-Ge(1)-P(2)	104.54(5)	96.91
P(3)-Ge(2)-P(4)	95.58(5)	96.81
P(1)-Ge(1)-Ge(2)-P(4)	41.81(5)	39.19

Table S2. Experimental and calculated chemical shifts (ppm) and coupling constants (Hz) for **7**:

	Expt. (d_8-toluene 298 K)	Calcd. (PBE1PBE/def2QVZ(Ge, P), 6-31G(d,p) (C, H)
P(1)	-27.1	-10.5
P(2)	-27.1	-11.5
P(3)	-110.4	-73.9
P(4)	+22.9	+46.3
P(1)/P(2)-P(3)	---	4.5/-7.6
P(1)/P(2)-P(4)	79.0	66.8
P(3)-P(4)	485.9	432.2

References:

[S1] R. A. Bartlett, M. M. Olmstead, P. P. Power, G. A. Sigel, *Inorg. Chem.* **1987**, *26*, 1941-1946.

[S2] CrysAlisPro, Agilent Technologies, Version 1.171.36.

[S3] R. C. Clark, J. S. Reid, *Acta Cryst.* **1995**, *A51*, 887-897.

[S4] (a) G. M. Sheldrick, *Acta Cryst.* **2015**, *A71*, 3-8. (b) G. M. Sheldrick, *Acta Cryst.* **2008**, *A64*, 112-122.

[S5] O. V. Dolomanov, L. J. Bourhis, R. J. Gildea, J. A. K. Howard, H. Puschmann, *J. Appl. Cryst.*, **2009**, *42*, 339-341.

[S6] Gaussian 09, Revision D.01, Frisch, M. J.; Trucks, G. W.; Schlegel, H. B.; Scuseria, G. E.; Robb, M. A.; Cheeseman, J. R.; Scalmani, G.; Barone, V.; Mennucci, B.; Petersson, G. A.; Nakatsuji, H.; Caricato, M.; Li, X.; Hratchian, H. P.; Izmaylov, A. F.; Bloino, J.; Zheng, G.; Sonnenberg, J. L.; Hada, M.; Ehara, M.; Toyota, K.; Fukuda, R.; Hasegawa, J.; Ishida, M.; Nakajima, T.; Honda, Y.; Kitao, O.; Nakai, H.; Vreven, T.; Montgomery, J. A., Jr.; Peralta, J. E.; Ogliaro, F.; Bearpark, M.; Heyd, J. J.; Brothers, E.; Kudin, K. N.; Staroverov, V. N.;

Kobayashi, R.; Normand, J.; Raghavachari, K.; Rendell, A.; Burant, J. C.; Iyengar, S. S.; Tomasi, J.; Cossi, M.; Rega, N.; Millam, J. M.; Klene, M.; Knox, J. E.; Cross, J. B.; Bakken, V.; Adamo, C.; Jaramillo, J.; Gomperts, R.; Stratmann, R. E.; Yazyev, O.; Austin, A. J.; Cammi, R.; Pomelli, C.; Ochterski, J. W.; Martin, R. L.; Morokuma, K.; Zakrzewski, V. G.; Voth, G. A.; Salvador, P.; Dannenberg, J. J.; Dapprich, S.; Daniels, A. D.; Farkas, Ö.; Foresman, J. B.; Ortiz, J. V.; Cioslowski, J.; Fox, D. J. Gaussian, Inc., Wallingford CT, 2009.

[S7] S. Grimme, *J. Comput. Chem.* **2006**, *27*, 1787-1799.

[S8] (a) A. D. McLean, G. S. Chandler, *J. Chem. Phys.* **1980**, *72*, 5639-5648. (b) K. Raghavachari, J. S. Binkley, R. Seeger, J. A. Pople, *J. Chem. Phys.*, **1980**, *72*, 650-654. (c) R. C. Binning Jr., L. A. Curtiss, *J. Comput. Chem.* **1990**, *11*, 1206-1216; (d) L. A. Curtiss, M. P. McGrath, J.-P. Blaudeau, N. E. Davis, R. C. Binning Jr., L. Radom, *J. Chem. Phys.* **1995**, *103*, 6104-6113.

[S9] NBO Version 3.1, E. D. Glendening, A. E. Reed, J. E. Carpenter, F. Weinhold,

[S10] (a) R. McWeeny, *Phys. Rev.*, **1962**, *126*, 1028-1034. (b) R. Ditchfield, *Mol. Phys.*, **1974**, *27*, 789-807. (c) K. Wolinski, J. F. Hilton, P. Pulay, *J. Am. Chem. Soc.*, **1990**, *112*, 8251-8260. (d) J. R. Cheeseman, G. W. Trucks, T. A. Keith, M. J. Frisch, *J. Chem. Phys.*, **1996**, *104*, 5497-5509.
