# Supplementary material for: Spontaneous Decomposition of an Extraordinarily Twisted and Trans‐Bent Fully‐Phosphanyl‐Substituted Digermene to an Unusual GeI Cluster
Source: Angew Chem Int Ed Engl. 2022 Aug 25;61(39):e202208851. doi: 10.1002/anie.202208851 (PMC9804623; doi:10.1002/anie.202208851)

checkCIF/PLATON report

Structure factors have been supplied for datablock(s) kji190022_face_abs

THIS REPORT IS FOR GUIDANCE ONLY. IF USED AS PART OF A REVIEW PROCEDURE FOR PUBLICATION, IT SHOULD NOT REPLACE THE EXPERTISE OF AN EXPERIENCED CRYSTALLOGRAPHIC REFEREE.

No syntax errors found. CIF dictionary Interpreting this report

Datablock: kji190022_face_abs

```
Bond precision:  C-C = 0.0102 A          Wavelength=1.54178

Cell:          a=14.8314(8)      b=17.8886(9)     c=29.3062(17)
               alpha=75.704(3)   beta=80.589(3)   gamma=69.962(3)
Temperature:   200 K

                Calculated                 Reported
Volume          7051.8(7)                  7051.8(7)
Space group     P -1                       P -1
Hall group      -P 1                       -P 1
Moiety formula  C72 H88 Ge2 P4 [+ solvent] C72 H88 Ge2 P4
Sum formula     C72 H88 Ge2 P4 [+ solvent] C72 H88 Ge2 P4
Mr              1222.53                    1222.48
Dx,g cm-3       1.151                      1.151
Z               4                          4
Mu (mm-1)       2.180                      2.180
F000            2576.0                     2576.0
F000'           2576.85
h,k,lmax        17,21,34                   17,21,34
Nref            25095                      23379
Tmin,Tmax       0.833,0.937                0.754,1.000
Tmin'           0.647

Correction method= # Reported T Limits: Tmin=0.754 Tmax=1.000
AbsCorr = MULTI-SCAN

Data completeness= 0.932          Theta(max)= 66.895

R(reflections)= 0.0748( 15121)    wR2(reflections)= 0.2301( 23379)

S = 1.041                  Npar= 1453
```

The following ALERTS were generated. Each ALERT has the format
test-name_ALERT_alert-type_alert-level.
Click on the hyperlinks for more details of the test.

Alert level A

```
PLAT029_ALERT_3_A _diffrn_measured_fraction_theta_full value Low .      0.932 Why?
```

Author Response: Crystal quality was compromised by the degradation caused by rapid solvent loss. This factor in addition to the dynamic disorder manifest in the structure and the disorder of the solvent molecules meant that a lot of high-angle reflections were not measurable.

```
PLAT910_ALERT_3_A Missing # of FCF Reflection(s) Below Theta(Min).        132 Note
```

Author Response: Crystal quality was compromised by the degradation caused by rapid solvent loss. This factor in addition to the dynamic disorder manifest in the structure and the disorder of the solvent molecules meant that a lot of high-angle reflections were not measurable.

Alert level B

```
PLAT911_ALERT_3_B Missing FCF Refl Between Thmin & STh/L=    0.597       1584 Report
```

Author Response: Crystal quality was compromised by the degradation caused by rapid solvent loss. This factor in addition to the dynamic disorder manifest in the structure and the disorder of the solvent molecules meant that a lot of high-angle reflections were not measurable.

Alert level C

```
PLAT220_ALERT_2_C NonSolvent   Resd 2  C   Ueq(max)/Ueq(min) Range        3.6 Ratio
PLAT242_ALERT_2_C Low    'MainMol' Ueq as Compared to Neighbors of        C76 Check
PLAT242_ALERT_2_C Low    'MainMol' Ueq as Compared to Neighbors of        C85 Check
PLAT242_ALERT_2_C Low    'MainMol' Ueq as Compared to Neighbors of       C103 Check
PLAT341_ALERT_3_C Low Bond Precision on  C-C Bonds ..............    0.01017 Ang.
PLAT906_ALERT_3_C Large K Value in the Analysis of Variance ......      5.470 Check
```

Alert level G

```
PLAT003_ALERT_2_G Number of Uiso or Uij Restrained non-H Atoms ...        156 Report
PLAT014_ALERT_1_G N.O.K.   _shelx_fab_checksum Found in CIF ......     Please Check
PLAT072_ALERT_2_G SHELXL First  Parameter in WGHT  Unusually Large       0.14 Report
PLAT154_ALERT_1_G The s.u.'s on the Cell Angles are Equal ..(Note)      0.003 Degree
PLAT187_ALERT_4_G The CIF-Embedded .res File Contains RIGU Records          1 Report
PLAT380_ALERT_4_G Incorrectly? Oriented X(sp2)-Methyl Moiety .....       C117 Check
PLAT606_ALERT_4_G Solvent Accessible VOID(S) in Structure ........          ! Info
PLAT802_ALERT_4_G CIF Input Record(s) with more than 80 Characters          1 Info
PLAT860_ALERT_3_G Number of Least-Squares Restraints ............        1210 Note
PLAT909_ALERT_3_G Percentage of I>2sig(I) Data at Theta(Max) Still        35% Note
PLAT913_ALERT_3_G Missing # of Very Strong Reflections in FCF ....          3 Note
PLAT941_ALERT_3_G Average HKL Measurement Multiplicity ...........        2.9 Low
PLAT978_ALERT_2_G Number C-C Bonds with Positive Residual Density.          0 Info
```

```
  2 ALERT level A = Most likely a serious problem - resolve or explain
  1 ALERT level B = A potentially serious problem, consider carefully
  6 ALERT level C = Check. Ensure it is not caused by an omission or oversight
 13 ALERT level G = General information/check it is not something unexpected

  2 ALERT type 1 CIF construction/syntax error, inconsistent or missing data
  7 ALERT type 2 Indicator that the structure model may be wrong or deficient
  9 ALERT type 3 Indicator that the structure quality may be low
  4 ALERT type 4 Improvement, methodology, query or suggestion
  0 ALERT type 5 Informative message, check
```

It is advisable to attempt to resolve as many as possible of the alerts in all categories. Often the minor alerts point to easily fixed oversights, errors and omissions in your CIF or refinement strategy, so attention to these fine details can be worthwhile. In order to resolve some of the more serious problems it may be necessary to carry out additional measurements or structure refinements. However, the purpose of your study may justify the reported deviations and the more serious of these should normally be commented upon in the discussion or experimental section of a paper or in the "special_details" fields of the CIF. checkCIF was carefully designed to identify outliers and unusual parameters, but every test has its limitations and alerts that are not important in a particular case may appear. Conversely, the absence of alerts does not guarantee there are no aspects of the results needing attention. It is up to the individual to critically assess their own results and, if necessary, seek expert advice.

Publication of your CIF in IUCr journals

A basic structural check has been run on your CIF. These basic checks will be run on all CIFs submitted for publication in IUCr journals (*Acta Crystallographica*, *Journal of Applied Crystallography*, *Journal of Synchrotron Radiation*); however, if you intend to submit to *Acta Crystallographica Section C* or *E* or *IUCrData*, you should make sure that full publication checks are run on the final version of your CIF prior to submission.

Publication of your CIF in other journals

Please refer to the *Notes for Authors* of the relevant journal for any special instructions relating to CIF submission.

PLATON version of 18/09/2020; check.def file version of 20/08/2020

Datablock kji190022_face_abs - ellipsoid plot

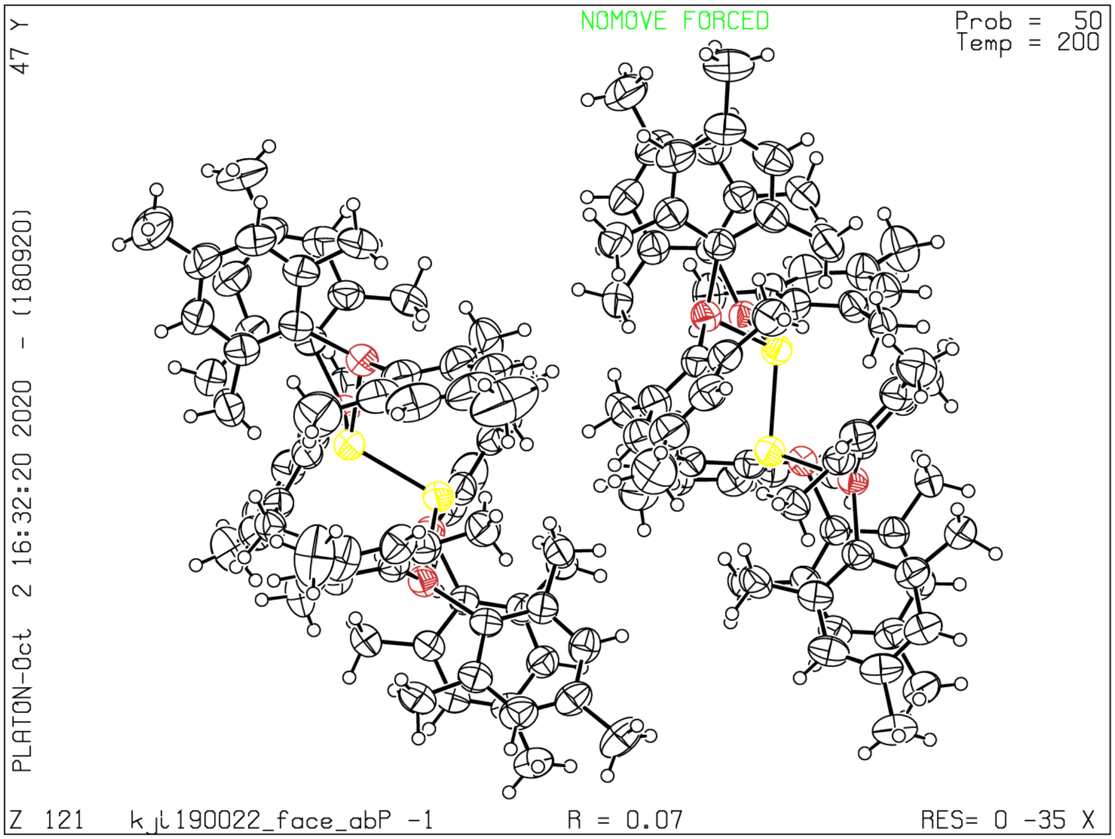

Supplement: Supplementary file 2 — Supporting Information [file ANIE-61-0-s004.pdf]
